# Fast, Accurate, and System-Specific Variable-Resolution Modeling of Proteins

**DOI:** 10.1021/acs.jcim.2c01311

**Published:** 2023-02-03

**Authors:** Raffaele Fiorentini, Thomas Tarenzi, Raffaello Potestio

**Affiliations:** † Department of Physics, University of Trento, via Sommarive 14, I-38123 Trento, Italy; ‡ INFN-TIFPA, Trento Institute for Fundamental Physics and Applications, via Sommarive 14, I-38123 Trento, Italy

## Abstract

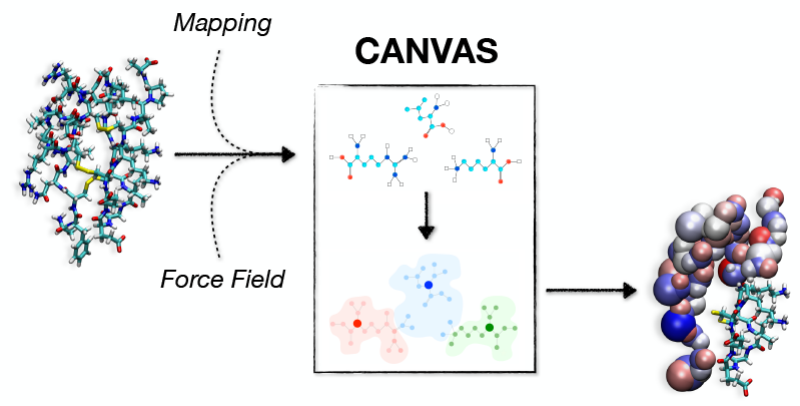

In recent years, a few multiple-resolution modeling strategies have been proposed, in which functionally relevant parts of a biomolecule are described with atomistic resolution, with the remainder of the system being concurrently treated using a coarse-grained model. In most cases, the parametrization of the latter requires lengthy reference all-atom simulations and/or the usage of off-shelf coarse-grained force fields, whose interactions have to be refined to fit the specific system under examination. Here, we overcome these limitations through a novel multiresolution modeling scheme for proteins, dubbed coarse-grained anisotropic network model for variable resolution simulations, or CANVAS. This scheme enables a user-defined modulation of the resolution level throughout the system structure; a fast parametrization of the potential without the necessity of reference simulations; and the straightforward usage of the model on the most commonly used molecular dynamics platforms. The method is presented and validated with two case studies, the enzyme adenylate kinase and the therapeutic antibody pembrolizumab, by comparing the results obtained with the CANVAS model against fully atomistic simulations. The modeling software, implemented in Python, is made freely available for the community on a collaborative github repository.

## Introduction

Steady improvements in high-performance computing hardware and molecular dynamics (MD) simulation software over several decades have ushered in impressive advancements in the computer-aided investigation of soft and biological matter systems, in particular, macromolecules of biological origin such as lipids, proteins, and nucleic acids.^1−3^ At the same time, a detailed modeling of molecular systems in which each atom is described as an interaction center often turns out to be inconvenient or even undesirable—on the one hand, because of major computing and data storage requirements and, on the other hand, because of the effort in analyzing the simulation outcome. To overcome both limitations, simplified, *coarse-grained* (CG) models,^4−9^ in which several atoms are lumped together in effective interaction sites, are frequently employed. CG models enable the simulation of larger systems over longer time scales, thanks to a smoother (free) energy profile and fewer degrees of freedom with respect to all-atom representations.

Coarse-grained models have been successfully employed for a number of biologically and pharmacologically relevant applications. These include the study of spontaneous protein–ligand binding,^10^ where both the macromolecule, the ligand, and the solvent are modeled in a coarse-grained fashion. The approach proved useful for the identification of binding pockets and the estimation of binding free energies on a number of systems; however, the simplified representation of the ligand requires a not-so-obvious new parametrization of the interactions and limits the distinction between similar molecules (as in the case of enantiomers).^10^ In addition, the employment of a coarse-grained solvent model limits the accuracy in the case where single water molecules are actively involved in the stabilization of the ligand in the binding site. Similarly, a number of coarse-grained force fields, including, among others, MARTINI,^11,12^ SIRAH,^13,14^ AWSEM,^15^ and Scorpion^16^ force fields, have been used to investigate protein–protein interactions, providing accurate results in terms of binding free energies. However, those models prevent an even coarser representation of the protein interfaces, which might be desirable in the case of very large protein assemblies; furthermore, they do not provide an accurate description of the system if specific atomistic details, possibly crucial for the properties or behavior of interest, are effectively integrated out in the low-resolution model.

Hence, whereas all-atom models provide the necessary accuracy at the expense of substantial computational resources, CG models enable a more efficient and intelligible representation of the system at the cost of losing possibly crucial detail. Although several problems in computational biophysics can be tackled with one of these two methods, many open questions remain that necessitate an approach at the interface between chemical accuracy and computational efficiency. In this regard, methods have been developed in which molecules described at different resolutions are simultaneously simulated within the same setup. Examples include the following: coupling of MARTINI with atomistic force fields;^17,18^ the simulation of atomistic proteins and nucleic acids in a multiresolution solvent with the SWINGER algorithm;^19,20^ and the simulation of soluble proteins with the PACE force field, which has been developed with the specific aim of coupling united-atom protein models with a coarse-grained solvent representation.^21,22^ Pushing the “resolution mix” even further, in some applications it might be desirable to couple different levels of detail within the same biomolecule to limit the computationally expensive high-resolution modeling to a subset of protein residues or nucleic acid base pairs. This approach was pioneered by the quantum mechanics/molecular mechanics (QM/MM) methods,^23−27^ which allow a connection between a small region where *ab initio* models are used, and a classical all-atom description in the remainder of the system. Along the same lines, several methodologies have been developed to couple atomistic and coarse-grained levels of resolution within the same simulation setup, and even within the same molecular structure. For example, in the Molecular Mechanics/Coarse-Grained (MM/CG) scheme developed in 2005 by Neri et al.,^28^ the atomistically detailed active site is incorporated into a coarse-grained Go̅-like model, which aims at reproducing the correct conformational fluctuations of the full protein.^29^ The MM/CG method was later tailored for the simulation of membrane protein/ligand complexes,^30^ and in the last version of the method, dubbed open-boundary MM/CG,^31^ the dual-resolution description of the protein is coupled with an adaptive multiscale model of the solvent, namely, the Hamiltonian adaptive resolution scheme (H-AdResS);^32,33^ in the latter, regions of different resolution are defined in the simulation box, allowing water molecules to change their resolution *on the fly* when diffusing from one region to the other. More recently, a similar method^34,35^ employed a high-resolution force field in small regions of a protein, most notably, the active site, while treating the remainder in a coarse-grained fashion, for example, as an elastic network model.^36^

Dual-resolution methods have been successfully applied for the study of several biological systems, including soluble^35^ and membrane proteins.^37−39^ However, the available approaches share some common shortcomings: first, the standard modeling of the CG region allows little flexibility in the choice of the CG sites; second, the CG region is usually defined *ad hoc*, and new mappings require a completely new reparameterization of the interactions; third, nonbonded interactions (such as electrostatics) are typically not taken into account in the CG model, thus preventing interactions between different structural domains that might come in close contact during the course of the simulation. CG models with an accurate description of electrostatics have been developed;^40−42^ however, in such cases, the protein is uniformly coarse-grained at a resolution intermediate between the atomistic and one-bead-per-amino acid one, thus limiting the level of coarse-graining and preventing a straightforward coupling between regions at different resolutions. These limitations hinder the applicability of standard multiple-resolution models, with detrimental consequences for the *in silico* investigation of proteins and their interactions.

In this work we propose a novel approach, dubbed coarse-grained anisotropic network model for variable resolution simulations, or CANVAS, which enables a fast parametrization of multiple-resolution models. The CANVAS strategy leverages the blurred and approximate nature of coarse-grained models to identify effective sites based on a user-provided input and determines the interactions among them based on the molecule’s structure and all-atom force field, making it unnecessary to run reference simulations. This strategy makes the parametrization of the model practically instantaneous and allows the modulation of the system’s resolution in a quasi-continuous manner across the structure, from all-atom to (very) coarse-grained. Most notably, the interaction between regions of the system at different resolutions (including the solvent) is accounted for and straightforward to set up, allowing the seamless implementation in standard MD software packages (e.g., GROMACS or LAMMPS).

The article is structured as follows: first, we describe in detail the CANVAS model, focusing on the construction of the multiple-resolution representation and on the parametrization of the interactions. A Methods section follows, providing the simulation details. The results of the validation of the CANVAS approach are then presented by comparing results from all-atom and multiscale simulations of two biomolecules, namely, the enzyme adenylate kinase and the IgG4 antibody pembrolizumab, each modeled with three resolution levels. Finally, conclusions and perspectives are discussed.

## The CANVAS Model

**Figure 1:**
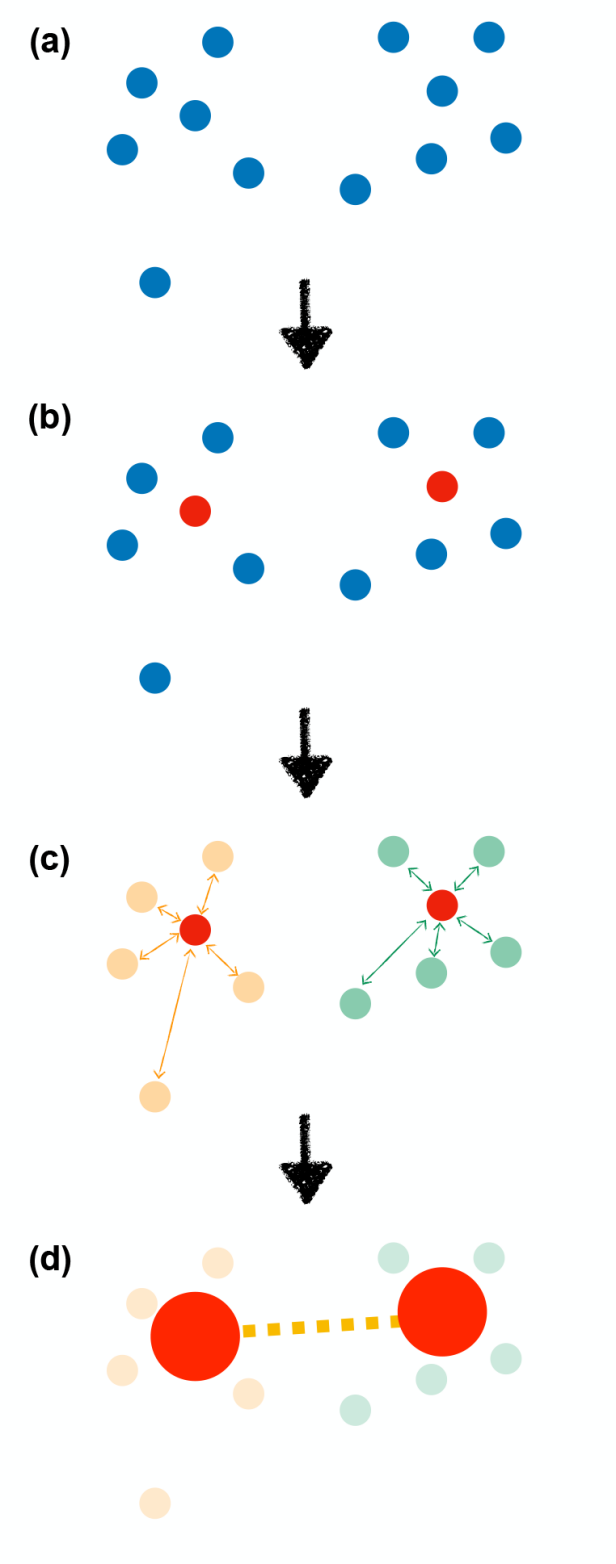
Scheme of the decimation process in the low-resolution part of the biomolecule. (a) Blue circles show all the atoms in the low-resolution part. (b) Choice of atoms that survive depicted in red. (c) Decimated atoms (light orange and light green) are mapped onto their closest survived atom in terms of Euclidean distance (orange and green arrows), according to the Voronoi tessellation. (d) Each survived atom, shown with a large red circle, is representative of the closest not-survived atoms mapped by it. A harmonic spring, depicted with a dashed yellow line, connects the neighboring survived atoms.

In the CANVAS approach to multiresolution protein modeling, a decimation mapping is implemented for the choice of the interactions sites:^9^ those atoms included in a user-defined list are retained, whereas the other ones are discarded. If all atoms of a given subregion of the molecule are retained, the high-resolution atomistic description is employed; in contrast, regions where atoms are removed are described at a varying level of detail. In lower-resolution regions, the physical properties of the survived atoms are modified so as to incorporate in effective interactions those atoms that have been integrated out (Figure 1). Specifically, each discarded atom is associated with the closest surviving one, and the properties of the latter are determined from those of the group of discarded atoms it represents.

The CANVAS model enables in principle a quasi-continuous modulation of the resolution of a protein or part of it, in that the detail of the representation can be gradually reduced from the all-atom level to a very coarse one, possibly lower than a few (from one to three) amino acids per bead; between the highest and lowest resolutions, an arbitrary number of intermediate levels are feasible. In the current implementation, we performed the choice of employing three levels of resolution:

- **all-atom (AT)**: the highest level of detail, where all the atoms of a given amino acid are retained;
- **medium-grained (MG)**: intermediate level of detail, where only the backbone atoms of an amino acid are retained, that is, the carbon alpha CA_mg_, the nitrogen *N*_mg_ of the amino group, the oxygen O_mg_, and the carbon C_mg_ of the carboxyl group;
- **coarse-grained (CG)**: the lowest level of resolution (in the applications presented here, only the C_α_ atoms of each CG residue are kept, dubbed CA_cg_.

The sets of protein residues modeled with an AT, MG, or CG detail are specified by the user and do not change during the simulation, that is, the biomolecule has a time-independent triple resolution. Table 1 summarizes the survived atoms in each region and their label.

**Table 1:** Description of Survived Atoms for Amino Acids (aa) for Each Level of Resolution

label	region	survived atoms per aa
at	fully-AT	all (CA_at_, N_at_, etc.)
mg	medium-grained	backbone (N_mg_, CA_mg_, C_mg_, O_mg_)
cg	coarse-grained	C_α_ (CA_cg_)

The first step of the model construction is to identify the region of the system where the chemical details play a crucial role such that no simplification of the atomistic description is desirable. Residues described at MG and CG resolutions can be either specified from the user or directly identified on the basis of the atomistic residues; in the latter case, the MG region is built by including those residues at a distance of 1 nm from the AT region, whereas the rest of the biomolecule is automatically assigned a CG representation.

**Figure 2:**
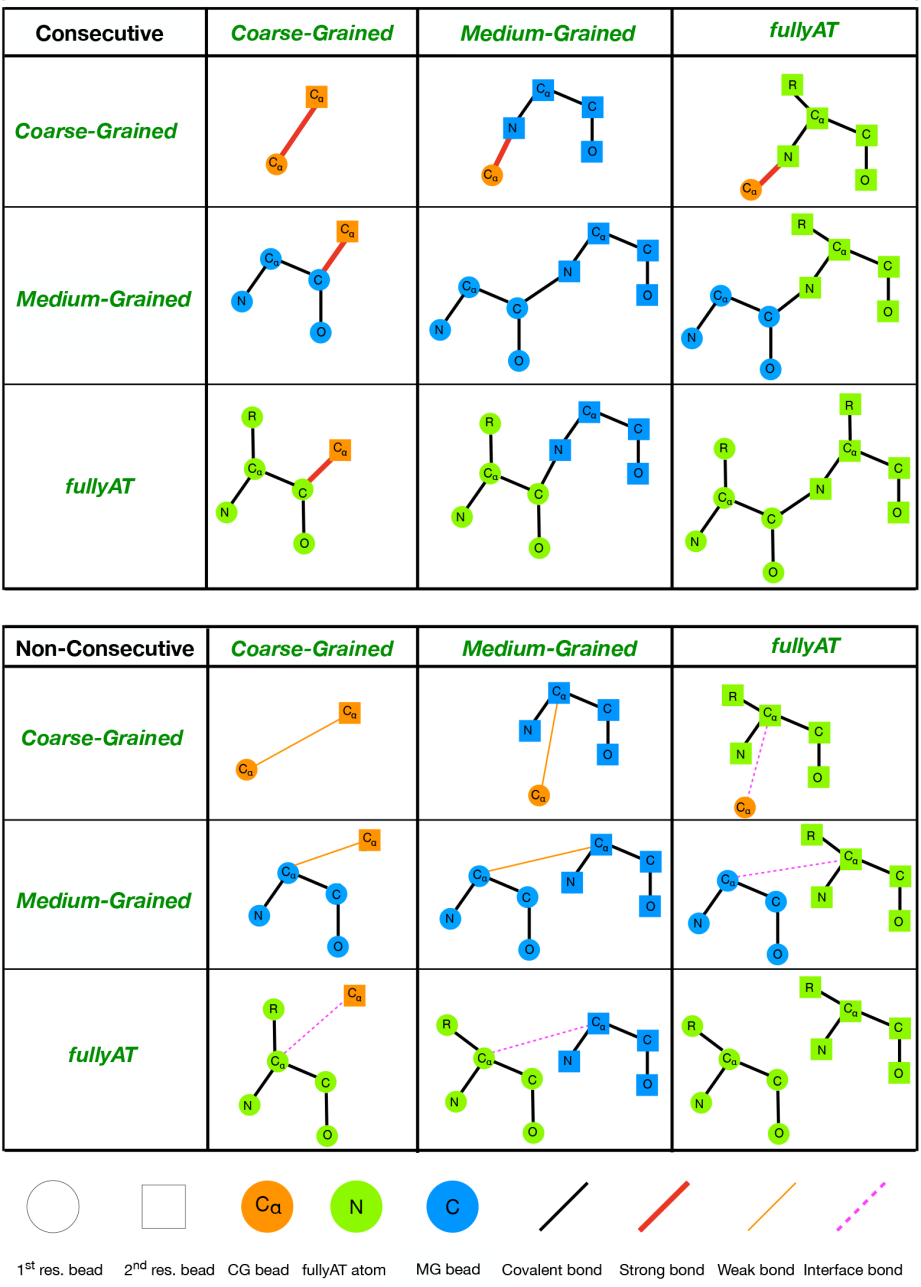
Schematic representation of the bonded interactions in the three regions at different resolutions. On the top of the figure, only consecutive residues are considered, and on the bottom, nonconsecutive ones. The atoms/beads that belong to the first residue are traced with a circle, whereas those that belong to the second residue are sketched with a square. R stands for the side chain. In the figure, hydrogen atoms are ignored for clarity, while being explicitly accounted for in the model. Bonded interactions are represented with different colors and thicknesses according to the spring constant.

The AT part is modeled through a standard atomistic force field (in the implementation discussed here, these are Amber99SB-ildn^43^ or CHARMM36m^44^), where the classical functional form and parametrization of the bonded and nonbonded interactions between atoms are employed. In the MG and CG domains, the potential energy is given by
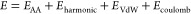
1The first term, *E*_AA_, corresponds to bonded interactions from the atomistic force field, namely, chemical bonds, angles, and proper/improper dihedrals:
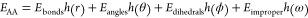
2Here, *h*(*r*), *h*(θ), *h*(ϕ), and *h*(ω) are Heaviside functions taking the value 1 if a bond, angle, dihedral, or improper dihedral exists in the atomistic force field for a couple, triplet, or quadruplet of survived atoms. Therefore, stretching, bending, and torsion potentials with their original equilibrium values are possible only if, respectively, the pair, triplet, and quadruplet of atoms (where at least a CG bead is involved) from the all-atom representation of reference are maintained in the MG and CG regions. The second term in eq 1, *E*_harmonic_, describes the bonded interactions between and within the low-resolution domains. The bonded connectivity and its parametrization are strictly dependent on the resolution levels employed and on the chemical nature of the retained sites, namely, on their *atom type*. In the current implementation, beads are connected by harmonic springs as schematically depicted in Figure 1d and described in detail in Figure 2. Specifically, the reference bond length corresponds to the distance between the two atoms/beads in the starting structure, whereas the value of the elastic constant depends on the nature of the bonded particles and their position along the sequence: (1) A stiff spring (*k*_b_) is employed for consecutive beads (red line of Figure 2); its value is 5 × 10^4^ kJ·mol^–1^·nm^–2^. (2) A weaker spring *k*_nb_ is used for nonconsecutive C_α_ beads (CA_cg_ – CA_mg_, CA_cg_ – CA_cg_, CA_mg_ – CA_mg_) whose distance in the reference (native) conformation lies below a fixed cutoff equal to 1.4 nm (orange line of Figure 2). Critically, the magnitude of *k*_nb_ depends on the distance *d* between the two C_α_ beads, farther CG units interacting through looser springs. The profile of *k*_nb_(*d*) was obtained through a statistical analysis performed over an ensemble of effective pair potentials acting among nonconsecutive C_α_ atoms in the pembrolizumab antibody; such potentials were extracted via direct Boltzmann inversion. See Section S1 in the Supporting Information for additional technical details. (3) A second weaker spring *k*_if_ is employed between an atomistic C_α_ and a CA bead (CA_at_ – CA_mg_ or CA_at_ – CA_cg_) if they do not belong to consecutive residues, and their distance in the reference conformation is less than a fixed cutoff equal to 1.4 nm (magenta dashed line of Figure 2). The recommended value of *k*_if_ is 50 kJ·mol^–1^·nm^–2^ to guarantee the appropriate degree of flexibility.

We stress that if the two survived atoms taken into account are connected by a covalent bond in the fully atomistic representation, the latter replaces *E*_harmonic_ (black line of Figure 2). Similarly, bending and torsion potentials with their original atomistic parametrization are maintained if the triplet and quadruplet of atoms (where at least a CG bead is involved) in the all-atom representation of reference are retained in the coarse regions. Rescaled nonbonded 1–4 interactions are introduced only in the AT region. In addition, to guarantee the correct degree of flexibility in multidomain proteins, no bond is introduced between those beads that are close in space in the starting configuration but belong to distinct structural domains; the latter can be defined either on the basis of the knowledge of the system or through appropriate algorithms developed to decompose protein structures in rigid subunits.^45,46^ The indices of the residues belonging to each domain are specified by the user in an optional input file.

**Figure 3:**
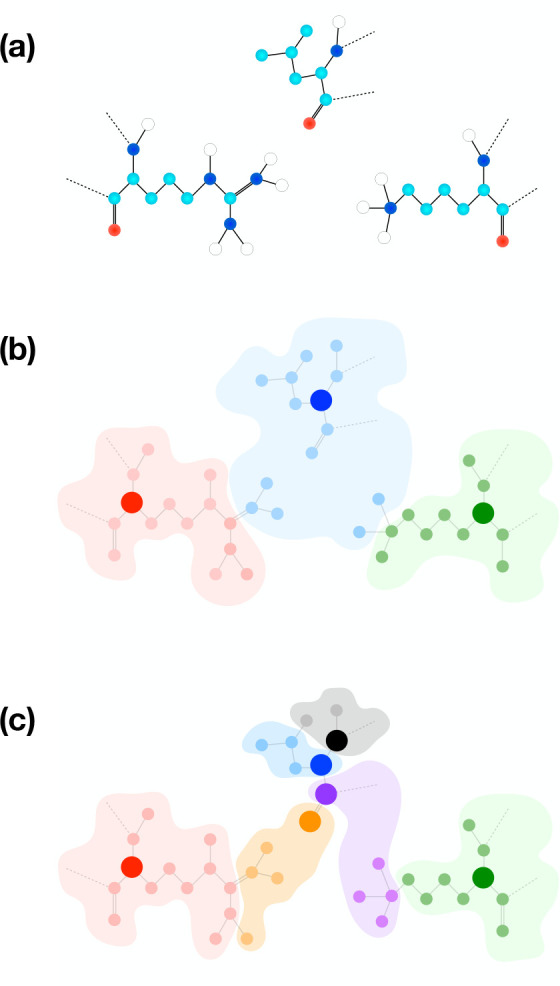
(a) Schematic representation of three amino acids: arginine, leucine, and lysine (from left to right). Dashed black lines represent the peptide bonds between two residues. The aliphatic hydrogens are not displayed for simplicity. (b) All three amino acids are modeled as CG, where only the C_α_ atom CA_cg_ (red, blue, and green bigger circles) are retained. The other atoms are decimated and mapped onto the closest survived atom (shown in pink, light blue, and light green). A bead is not necessarily representative of atoms belonging to the same residue since the grouping criterion is merely based on euclidean distance. (c) Arginine and lysine are modeled in CG (red and green bigger circles), whereas the leucine is described in MG (CA_mg_ in blue, N_mg_ in black, C_mg_ in violet, and O_mg_ in orange).

Finally, *E*_VdW_ and *E*_coulomb_ in eq 1 are the van der Waals and Coulomb nonbonded contributions to the potential energy between nodes. For the AT region, standard force-field parameters are taken, whereas in the MG and CG regions, the charge and Lennard-Jones parameters of each bead are computed from the average properties of the neighboring atoms, as defined through a procedure akin to a Voronoi tessellation.^47−49^ First, a Voronoi cell is defined by associating the decimated atoms (blue circles of Figure 1b) to the closest survived atom (in terms of Euclidean distance ), which is now treated as a CG bead (Figure 1c and Figure 3b,c). We emphasize that because a geometric criterion is employed to group atoms, the resulting bead is representative of atoms that could also belong to separate residues, as schematically shown in Figure 3. For this reason, the protein’s starting structure plays a relevant role in the Voronoi tessellation because the relative orientation of side chains might influence the construction of the cells. Therefore, it is important that the structure employed as a reference for coarse graining is minimized and equilibrated. For the same reason, the Voronoi tessellation-based coarse-graining procedure is strongly dependent on the starting structure, and we can expect the relative arrangements of secondary elements to be preserved during the simulation. If conformational changes are desirable, a careful distribution of the different degrees of resolution along the structure is required, and a more informed partition of the system should be done with explicit input from the user.

After the definition of the Voronoi cells, nonbonded potential parameters are computed for each CG bead. Specifically, for a mapping that retains *N* atoms out of *n*: (1) The charge *Q*_*I*_ is defined as the algebraic sum of the charges *q*_*i*_ of the atoms it represents:
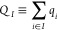
3(2) The diameter σ_*I*_ is twice the gyration radius *R*_g_:
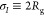
4where
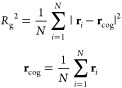
Here, **r**_*i*_ represents the coordinates of each atom, whereas **r**_cog_ corresponds to the coordinates of the center of geometry of the group. (3) ϵ_*I*_ is the geometric average of the ϵ_*i*_ values of the atoms it represents:
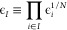
5

As opposed to the network of bonded interactions, where a predefined set of parameters is employed, the nonbonded part is automatically constructed on the basis of the properties of the retained sites, independently of the level of resolution and the bonded connectivity between them. The combination rule used to determine, from these parameters, Lennard-Jones interactions between CG beads is the same as the one employed by the atomistic force field in the high-resolution region; namely, it is based on the Lorentz–Berthelot rules for both the Amber and CHARMM force fields. In addition, in the case of interactions between nonconsecutive coarse-grained sites, nonbonded interactions are fully accounted for, whereas nonbonded interactions are switched off in the case of bonds involving atoms in the high-resolution region, as in the standard atomistic description.

We stress that because CG beads in the CANVAS representation may not be representative of a single residue, a direct residue-based analysis can not be performed. This is a specific feature of the CANVAS approach: the latter, in fact, was conceived to be easily generalized to very coarse mappings, where one bead is representative of more than one residue, or for inhomogeneous mappings, where the retained low-resolution sites are distributed throughout the protein independently from the residue at which they originally belong (so that some residues might be represented by one or more beads, whereas others might be discarded completely). In such a case one can use mappings that are different from the intuitive, chemistry-based ones, but that are the most efficient in preserving the information contained in the all-atom protein representation.^9,50^

The code and examples of input files for simulating a system with the CANVAS model are freely available at https://github.com/potestiolab/canvas. The code consists of two Python scripts: the first one (*block.py*) has the purpose of creating the list of survived atoms with their relative labels (AT, MG, or CG); the second script (*CANVAS.py*) returns the input files needed for simulating a solvated biomolecule in LAMMPS or GROMACS, according to the choice made by the user. The mandatory arguments for the successful execution of the code are the list of survived atoms, the coordinate file (.gro) and the topology file (.top) of fully atomistic representation. A detailed description of the other parameters (mandatory and optional) and a tutorial for the construction and simulation of a CANVAS model, starting from the atomistic representation, are available on the same repository.

## Materials and Methods

The two systems employed in the present work as a test bed for the CANVAS model are the enzyme adenylate kinase^51−53^ and the antibody pembrolizumab.^54^

**Figure 4:**
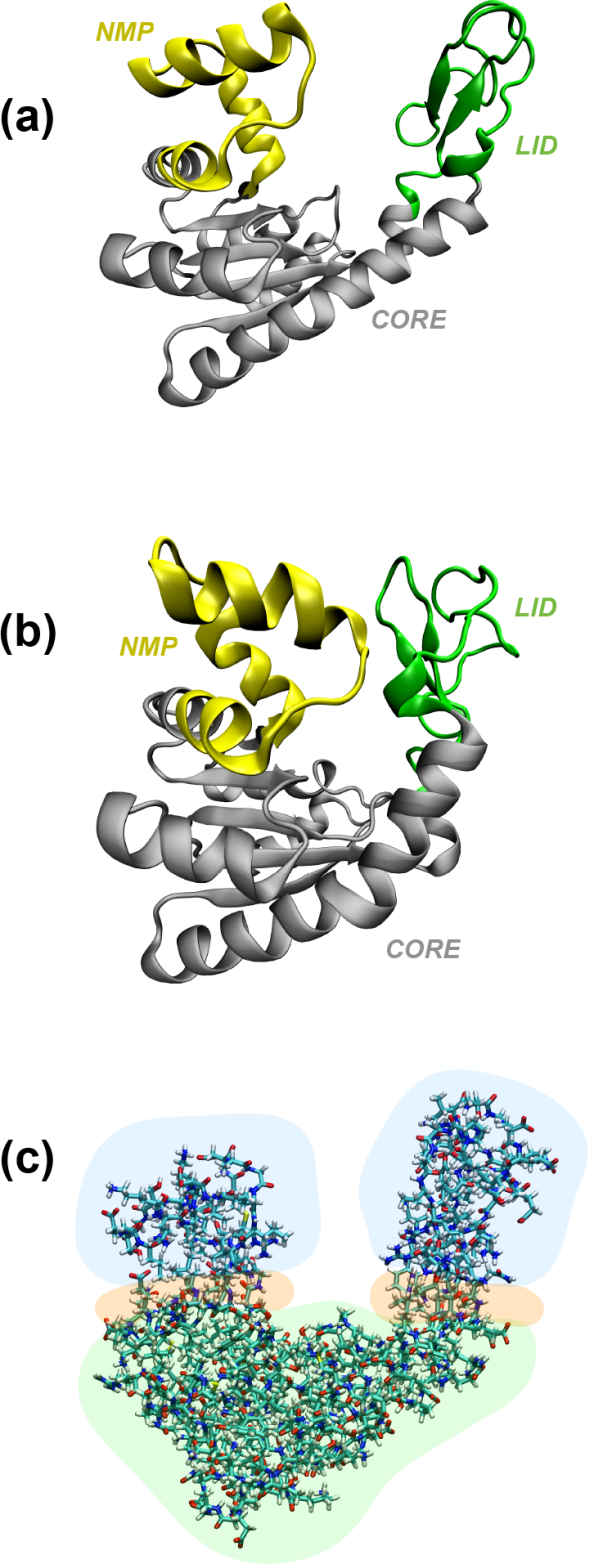
Fully atomistic representation of ADK. In particular, (a) and (b) show the open and compact conformation of the protein, respectively, in terms of secondary structure. LID, NMP, and CORE domains are depicted in green, yellow, and gray. (c) displays a schematic representation of the reference structure of ADK before conversion from the all-atom representation to the CANVAS one. Specifically, the CORE of the protein, modeled atomistically, is depicted in green; the part that is described in MG is shown in orange; the remainder, which is going to be coarse-grained, is shown in blue.

Adenylate kinase (ADK) plays a critical role in maintaining the energetic balance in the cell, interconverting adenosine diphosphate (ADP) molecules into adenosine monophosphate (AMP) and adenosine triphosphate (ATP).^55^ The structure of ADK can be partitioned in three domains, called the CORE, LID, and NMP, and two distinct nucleotide binding sites, as shown in Figure 4a,b.

**Figure 5:**
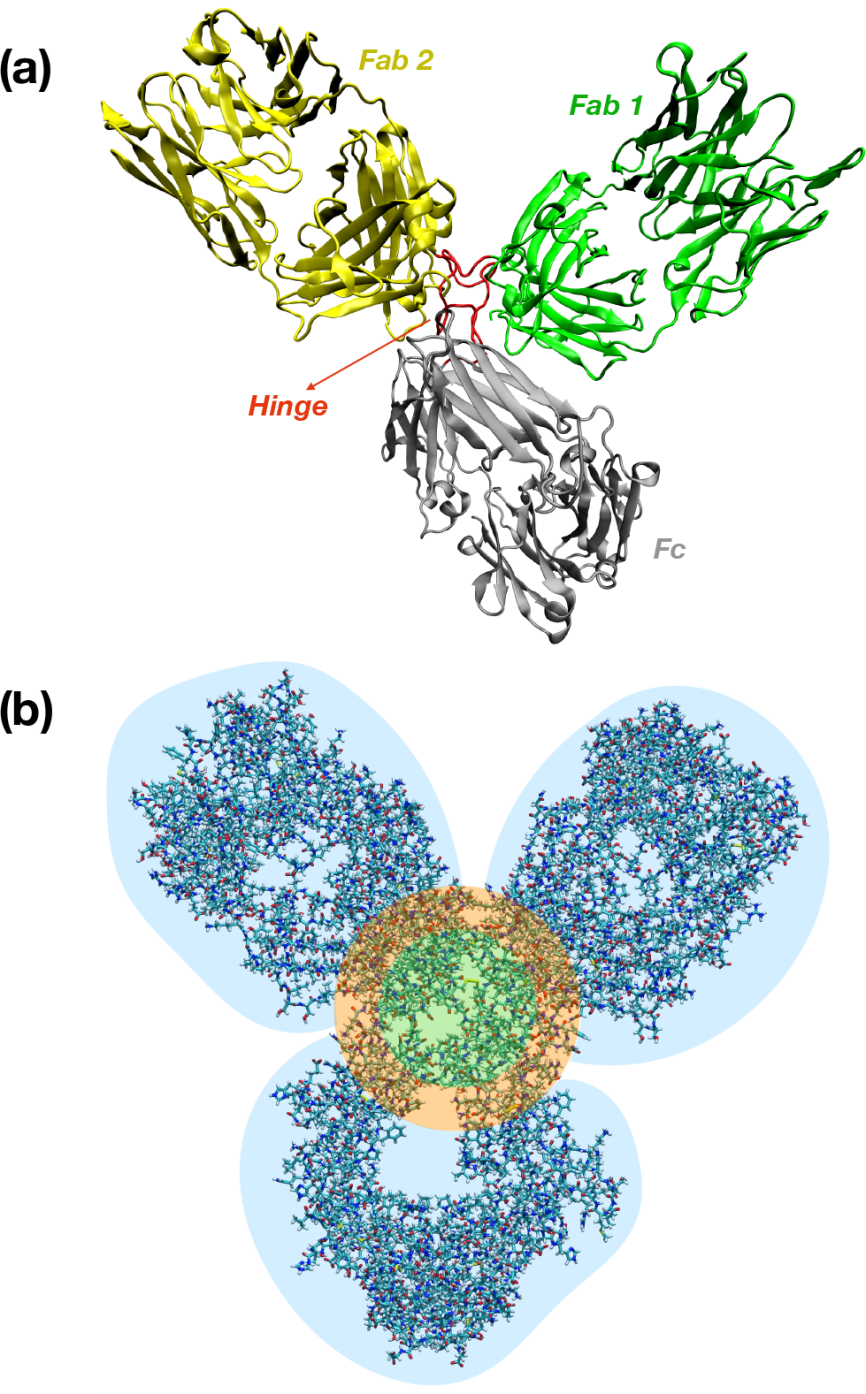
(a) Graphical representation of the crystallographic conformation of pembrolizumab in terms of secondary structure. Fab 1, Fab 2, Fc, and the hinge are depicted in green, yellow, gray, and red, respectively. (b) Schematic representation of the 4_A_ reference conformation of pembrolizumab before conversion from the all-atom to the multiresolution. In particular, in green is depicted the hinge of the biomolecule, modeled atomistically; in orange is shown the part that is going to be described as MG; the remainder, shown in blue, is going to be represented as CG.

The second system used here as a test case, pembrolizumab, is a humanized IgG4 antibody consisting of four chains, covalently bound by disulfide bonds (Figure 5a). Pembrolizumab—which is the generic name for the trade drug name Keytruda—is currently used in immunotherapy as an anticancer drug.^56^ Its antigen is the programmed cell death protein 1 (PD-1), expressed on the membrane of T cells, B cells, and natural killer cells; the formation of the high-affinity complex between the antibody and its antigen prevents the binding of PD-1 with the programmed cell death receptor ligands PD-L1 and PD-L2, which would lead to a suppression of the antitumor activity of T cells.^57^

The reference structures employed for the construction of the multiscale models were obtained from equilibrated all-atom simulations. Specifically, the crystallographic ADK structure (PDB ID: 4AKE) was solvated in an aqueous solution at 0.15 M NaCl concentration; after energy minimization, the system was equilibrated for 125 ns in the NPT ensemble, using the Parrinello–Rahman barostat^58^ with a time constant of 2.0 ps at 1 bar and the Langevin thermostat^59^ to keep the temperature at 300 K. The all-atom simulation was extended for 500 ns, on which the analyses were performed. For the CANVAS simulation, the equilibrated structure was placed in a cubic simulation box of 9.1 nm per side and solvated in an aqueous solution at 0.15 M NaCl concentration.

The reference structures of pembrolizumab are given by the representative conformations sampled from four all-atom 500 ns long simulations of the antibody in the apo form, after clustering the frames on the basis of their structural similarity. Each of these atomistic simulations was started from the PDB crystallographic structure of the deglycosilated antibody (PDB ID: 5DK3) after modeling of the missing residues; for more details on the all-atom simulation protocol, the reader is referred to ref (60). A CANVAS simulation is started from each representative conformation of the antibody, for a total of six different runs; this choice is dictated by the large conformational variability of the molecule, and the peculiar properties of each conformational basin. The CANVAS models of the representative structures are placed in a cubic simulation box of 17.9 nm per side and are solvated in a 0.15 M NaCl aqueous solution.

For both ADK and pembrolizumab, the force field employed was Amber99SB-ildn,^43^ and the water model was TIP3P.^61^ Furthermore, for the sake of assessing the validity of the approach independently of the specific all-atom force field employed, 10 ns long CANVAS simulations of ADK were performed with the Charmm36m force field, using MD software programs GROMACS and LAMMPS; the results of these tests are provided as Supporting Information in Figure S2. CANVAS systems were prepared starting from the representative structures obtained from the atomistic simulations, after energy minimization with the steepest descent algorithm and 100 ps of NVT equilibration. The temperature is kept constant at 300 K by means of the Langevin thermostat.^59^ In the NPT production run, the Parrinello–Rahman barostat is employed, as described above. The integration step is 2 fs. The calculation of electrostatic interactions is performed in all cases by using the reaction-field method^62,63^ with a dielectric constant of ϵ = 80 and a cutoff of 2.5σ_max_; here, σ_max_ is the maximum value of σ among all the beads of the system. To validate the choice of the AMBER force field in combination with the reaction-field method making use of the previous set of parameters, we also performed an atomistic MD simulation using PME for the description of electrostatic interactions, with a dielectric constant ϵ = 1 and a cutoff of 1.0 nm. The comparison is performed in terms of RMSF between the two all-atom trajectories of ADK, as shown in Figure S3. We observed that the trends of fluctuations are consistent with each other, providing comforting evidence that the AMBER model can be safely employed with a reaction field. The SETTLE^64^ and RATTLE^65^ algorithms for rigid water and rigid bonds containing hydrogen were used. The length of the CANVAS simulations is 500 ns for ADK and 200 ns for each antibody system. All simulations were carried out with GROMACS 2018.^66^ We stress here that the usage of an explicit solvent, while guaranteeing the highest level of accuracy of the model in the atomistic region, makes the computational cost of the simulation essentially identical to that of a fully atomistic model.In Table 2 we provide a quantitative comparison of the performance of 500 ns long ADK simulations run on a 48-cores single node. These show how the CANVAS simulation is slightly faster (about 1.05 times) than the atomistic one when using the reaction-field electrostatic method and same cutoff. Moreover, as expected, the all-atom simulation employing the reaction field is faster—about twice as fast—than the corresponding one when using PME. One of the long-term targets in the development of variable-resolution models is the boost of computational efficiency through the reduction of the number of model particles; here, however, we apply the multiscale representation for the biomolecule alone because the combined usage of multiple-resolution models of the protein *and* of the solvent would lead to ambiguities in the validation and in interpretation of the outcomes. The usage of CANVAS in combination with computationally efficient models of the solvent (e.g., implicit solvent^67,68^ or adaptive resolution simulation schemes^32,33,69^) will be the object of future work.

**Table 2:** Comparison of the Time Performance for ADK Simulations Run on 48 Cores, Single Node, for Different Electrostatic Methods, Interaction Cutoffs, and Resolution^a^

method	resolution	cutoff [nm]	performance
reaction field	all-atom	1.000	87.90 ns/day
reaction field	all-atom	1.698	32.14 ns/day
reaction field	CANVAS	1.698	33.75 ns/day
PME	all-atom	1.000	43.04 ns/day
PME	all-atom	1.698	19.17 ns/day

a As expected, those employing the PME are about twice as slow as those employing the reaction-field (RF) method for all-atom simulation and cutoff of 1.0 nm. The CANVAS simulation is slightly faster than the all-atom one when using the RF method and a cutoff of 1.698 nm. The latter value corresponds to 2.5σ_max_ for the ADK starting configuration when constructing the CANVAS model.

The analysis of fluctuations was performed with the VMD molecular visualization program.^70^ In particular, the root-mean-square deviation (RMSD) was computed through the *RMSD Trajectory Tool* considering the sole C_α_ atoms. The root-mean-square fluctuations (RMSF) were computed by means of an in-house tkl script. The radii of gyration were computed with *gmx gyrate*, whereas the solvent-accessible surface area was computed with *gmx sasa*. The principal component analysis and the calculation of the root-mean-square inner product (RMSIP)^71^ between the essential subspaces from atomistic and CANVAS simulations were performed with the Python module MDAnalysis. The calculation of the electrostatic potential was performed with the online adaptive Poisson–Boltzmann solver (APBS)^72^ after the creation of an input PQR file that, in the case of the multiscale model, includes the radii and charges as computed with the CANVAS protocol. Protein visualization and rendering was performed with VMD,^70^ whereas the plots were created with Xmgrace and Python libraries.

## Results and Discussion

In this section we compare results from the atomistic and CANVAS simulations for both ADK and pembrolizumb to assess the validity of the proposed multiscale model. In the case of pembrolizumab, the comparison is performed between the six CANVAS simulations and the corresponding ensembles of structurally homogeneous configurations obtained through a clustering of all-atom simulation frames; see Tarenzi et al.^60^

### Adenylate Kinase

The ADK protein exists in two main conformations, required for the catalytic activity of the enzyme: a fully open one, where the LID and the NMP domains are separated from each other, thus exposing the binding site; and a closed one, which is stabilized by the presence of the substrate and allows for the enzymatic reaction to take place.^73^ In the all-atom simulation, ADK samples both the open conformation, which corresponds to the starting structure (Figure 4a), and a more compact one (Figure 4b), where the distance between the LID and NMP arms is substantially reduced. This partially closed conformation of ADK in the apo state was already observed experimentally^74^ and in previous MD simulation studies.^75,76^ However, we do not observe a complete transition between the open and fully closed states, as expected from the absence of the substrate and from the long time scale of the process (on the order of μs to ms^77^); indeed, the computed distance between the C_α_ atoms of residues A55 and V169, previously used to discriminate the two conformational states both in experiments and simulations,^78^ is consistent with the open state for the whole duration of the trajectory (Figure S4 in the Supporting Information).

**Figure 6:**
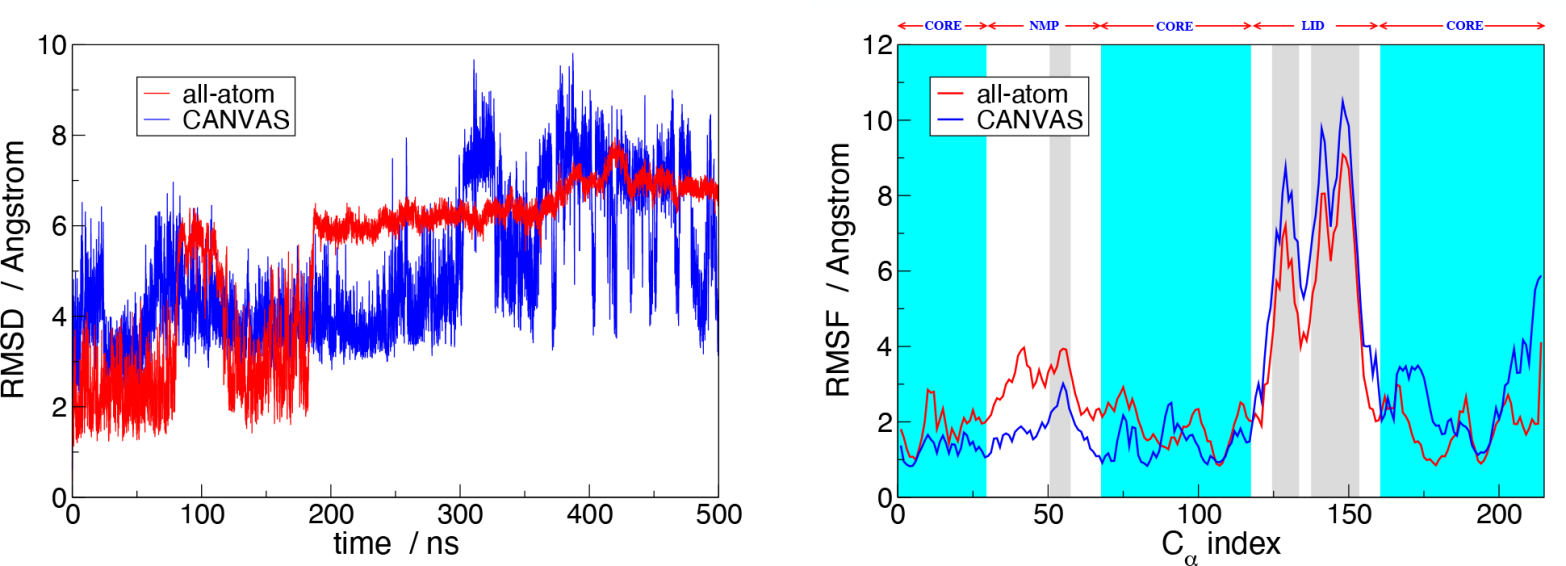
(a) RMSD of all ADK C_α_ comparing the all-atom simulation (red line) and the CANVAS one (blue line). The presence of two different states, one corresponding at about 3 Å and the second one close to 6 Å, are indicative of open and compact conformations, respectively. (b) RMSF for each C_α_ of ADK from the all-atom simulation (red line) and the CANVAS one (blue line). The cyan area corresponds to the CORE domain, which is described atomistically, whereas the gray and white regions correspond to the parts of the system (LID and NMP domains) modeled in CG and MG, respectively. Videos of the atomistic and CANVAS trajectories are provided on the Zenodo repository 10.5281/zenodo.7225086.

The evolution of the protein between the two aforementioned conformations can be quantified during the simulation in terms of the RMSD of all C_α_ atoms with respect to the initial frame, which corresponds to the equilibrated structure of ADK in the NPT ensemble (Figure 4a). Because the latter is in the open conformation, higher RMSD values are indicative of closer structures. The resulting plot is shown with a red line in Figure 6a. As expected, two states are clearly visible: one corresponding to 3 Å and the second one around 6 Å. The compact conformation (higher RMSD values) is attained for a few nanoseconds after 80 ns, it reappears subsequently after 200 ns and remains there until the end of the simulation.

Consistent with the previous analysis, the red line of Figure 6b shows the RMSF for each C_α_, computed with respect to the average structure: we notice that the atoms constituting the protein arms, that is, the LID and NMP domains (indices 118–160 and 30–67) have wider fluctuations with respect to the CORE.

Because the open/closed transition is determined by the relative orientation of LID and NMP with respect to the CORE, the latter is modeled at high resolution in the CANVAS simulation, with the aim of retaining a realistic degree of flexibility of the hinge. In contrast, the LID and the NMP domains are described using two levels of resolution, that is, MG and CG. We recall that all residues whose distance is less than 1 nm with respect to the closest all-atom residues are described as MG to guarantee a *smooth* transition between the highest and lowest levels of resolution. A schematic representation is shown in Figure 4c.

**Figure 7:**
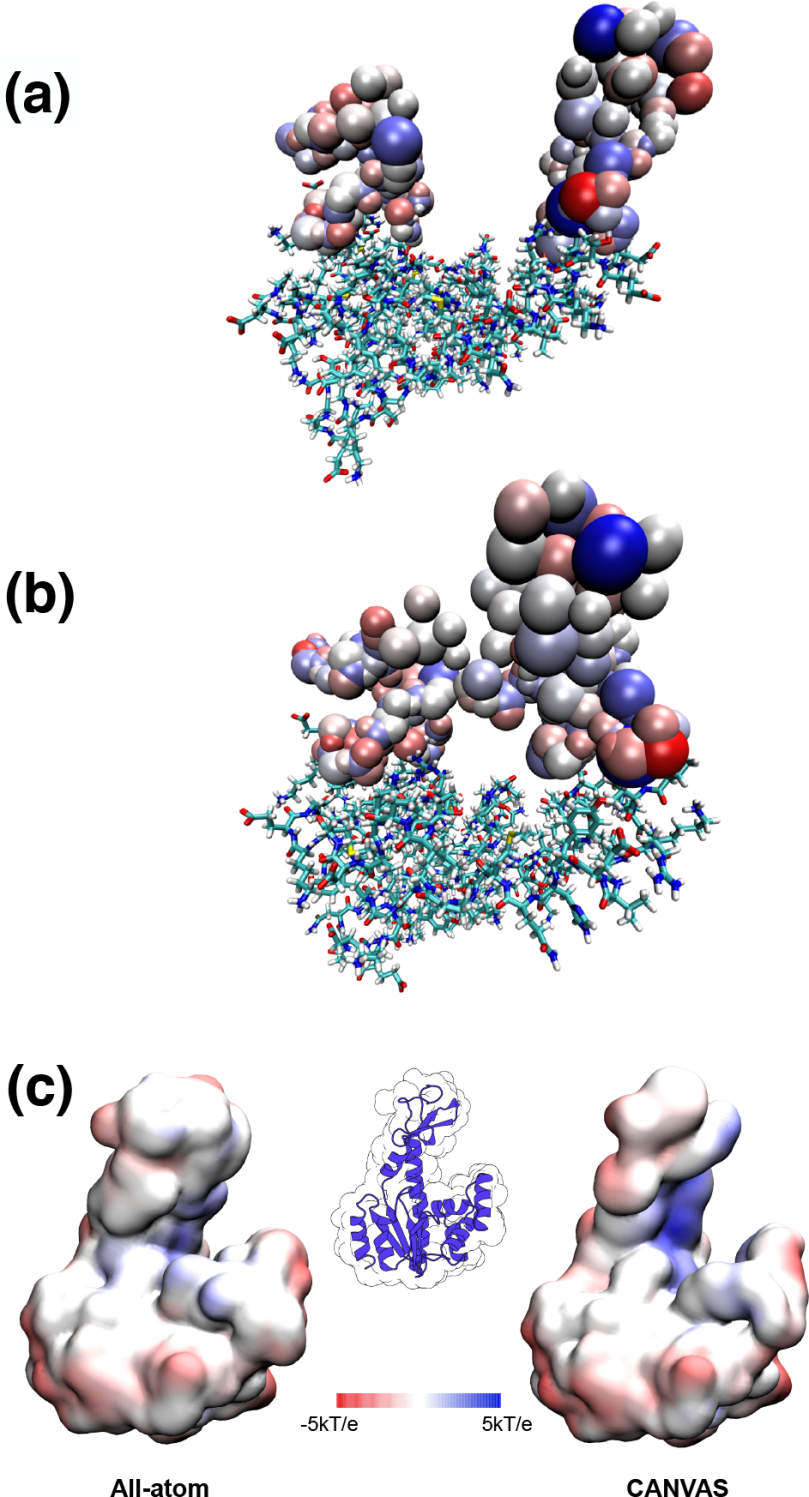
CANVAS representations of ADK, where the all-atom region is described in licorice and the MG/CG beads as VdW spheres. The diameter of each bead is given by the value of σ, whereas its color is dependent on the value of the charge: white spheres are indicative of neutral charge, whereas blue and red beads correspond to positive and negative charges, respectively. (a) shows the open conformation of the protein, whereas (b) shows the more compact one. (c) displays the electrostatic potential calculated with the APBS for the all-atom and CANVAS representations of the starting ADK structure, mapped on the protein surface.

The CANVAS simulation shows two main protein conformations, analogous to the all-atom simulation: the open one, as depicted in Figure 7a, and the compact one as displayed in Figure 7b. The interconversion between the two conformations is monitored, analogous to the fully atomistic simulation, by calculating the RMSD of the C_α_ atoms (CA_at_, CA_mg_, CA_cg_) with respect to the reference frame. The resulting curve is shown in blue in Figure 6a. The comparison between the all-atom and multiresolution RMSD shows that the CANVAS model reproduces well the conformational changes observed in the fully atomistic system, allowing the protein to transition between the two basins more frequently than the all-atom reference. To assess whether the two sampled states are structurally similar in both simulations, we performed a clustering analysis on the all-atom and CANVAS trajectories, using the RMSD with respect to the starting structure as a distance measure. From the two clusters obtained (corresponding to the fully open and to the compact conformations), the central structures are extracted; representative conformations belonging to the same state are then compared between the atomistic and multiscale cases (Figure S5), and the RMSD value between them was calculated. The resulting RMSD values are 3.7 Å for the open conformations and 5.5 Å for the compact ones; a visual inspection of the representative structures reveals that these deviations are mostly limited to the flexible and disordered regions of the protein, whereas the overall conformational state is the same in the atomistic and multiscale case. Conversely, the comparison of closed and open structures shows larger deviations: the RMSD value between the open atomistic and CANVAS compact representative conformations is 7.4 Å, whereas the RMSD value is 5.8 Å when comparing the compact atomistic and CANVAS open representative conformations. Next, we looked into the fluctuation of each C_α_ in the all-atom part and each CA bead (CA_mg_, CA_cg_) in the MG and CG ones (whose position is the same for the corresponding C_α_ atoms in the all-atom representation), as depicted by the blue line in Figure 6b. Also in this case, for both all-atom and lower-resolution regions the fluctuations of C_α_ atoms are comparable to those from the atomistic simulation. The comparison of fluctuations has also been performed independently on the sets of frames extracted from the atomistic and CANVAS trajectories after the RMSD-based clustering; the resulting RMSF is plotted in Figure S6 of the Supporting Information, and it shows a similar trend in the two cases.

As explained in the description of the model, the values of *Q*, σ, and ϵ for each low-resolution bead are different depending on the number and type of atoms that are mapped onto it. Figure 7 shows the two conformations where each CG bead is colored according to its charge, and whose size is based on the σ values. The partial charges assigned to each MG and CG bead, in addition to those assigned to each atom by the atomistic force field, were used to compute the electrostatic potential with the APBS.^72^ The protein surface, colored according to the mapped potential, is represented in Figure 7c for both the fully atomistic case and the CANVAS case; the comparison shows that the electrostatic patches are conserved in the multiresolution representation.

**Figure 8:**
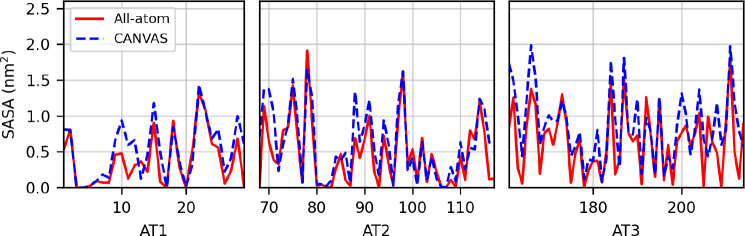
Per residue values of SASA, computed for the atomistic region of the ADK. The AT region is composed of three segments of consecutive residues, denoted AT1, AT2, and AT3.

To check the accuracy in the description of the AT region in the CANVAS model, we computed the average solvent-accessible surface area (SASA) for each atomistic residue, comparing the results to the values obtained from the atomistic simulation (Figure 8). The results are in good agreement; the slightly larger SASA values for some residues in the CANVAS simulation might be ascribed to the fact that in the fully atomistic case the protein spends a larger portion of the trajectory in the compact state, where the solvent accessibility of a number of residues is reduced.

### Pembrolizumab

In Tarenzi et al.,^60^ four all-atom simulations have been performed starting from the PDB crystallographic structure of the full-length antibody, for a total simulation time of 2 μs. The antibody conformations sampled from the MD simulations are grouped into six clusters on the basis of their structural similarity. The representative structures of the different clusters are shown in Figure S7 and labeled 0_A_, 1_A_, 2_A_, 3_A_, 4_A_, and 5_A_ according to the increasing value of the protein’s average radius of gyration. The conformations differ mainly in the relative orientation of the Fab and Fc domains, which can get in close contact thanks to the flexibility of the hinge region; the latter includes two 18 residue long disordered segments, bridged by two disulfide bonds.

The six representative pembrolizumab structures are taken as starting conformations for six CANVAS simulations. Because the variation in the relative arrangement of Fab1, Fab2, and Fc domains is made possible by the disordered hinge region, the latter is described atomistically, whereas the three large domains are modeled with lower levels of detail. In particular, those residues whose distances are less than 1 nm with respect to the closest fully atomistic ones are described as MG, whereas the rest is represented as CG. A schematic representation is given in Figure 5b.

Deviations from the starting structure along the simulations are plotted in Figure S8 in terms of RMSD and compared to the average RMSD of the atomistic frames falling within the same conformational cluster. Both atomistic and CANVAS deviations were computed with respect to the same reference structure. For the majority of the conformational clusters, the RMSD values from the CANVAS simulation fall within the error bar of the atomistic reference. However, Figure S8 suggests also that the CANVAS representation of pembrolizumab is slightly more rigid than the fully atomistic case; conversely, the atomistic conformations falling within the same cluster appear more heterogeneous, hence their largest values of RMSD with respect to the representative structures.

**Figure 9:**
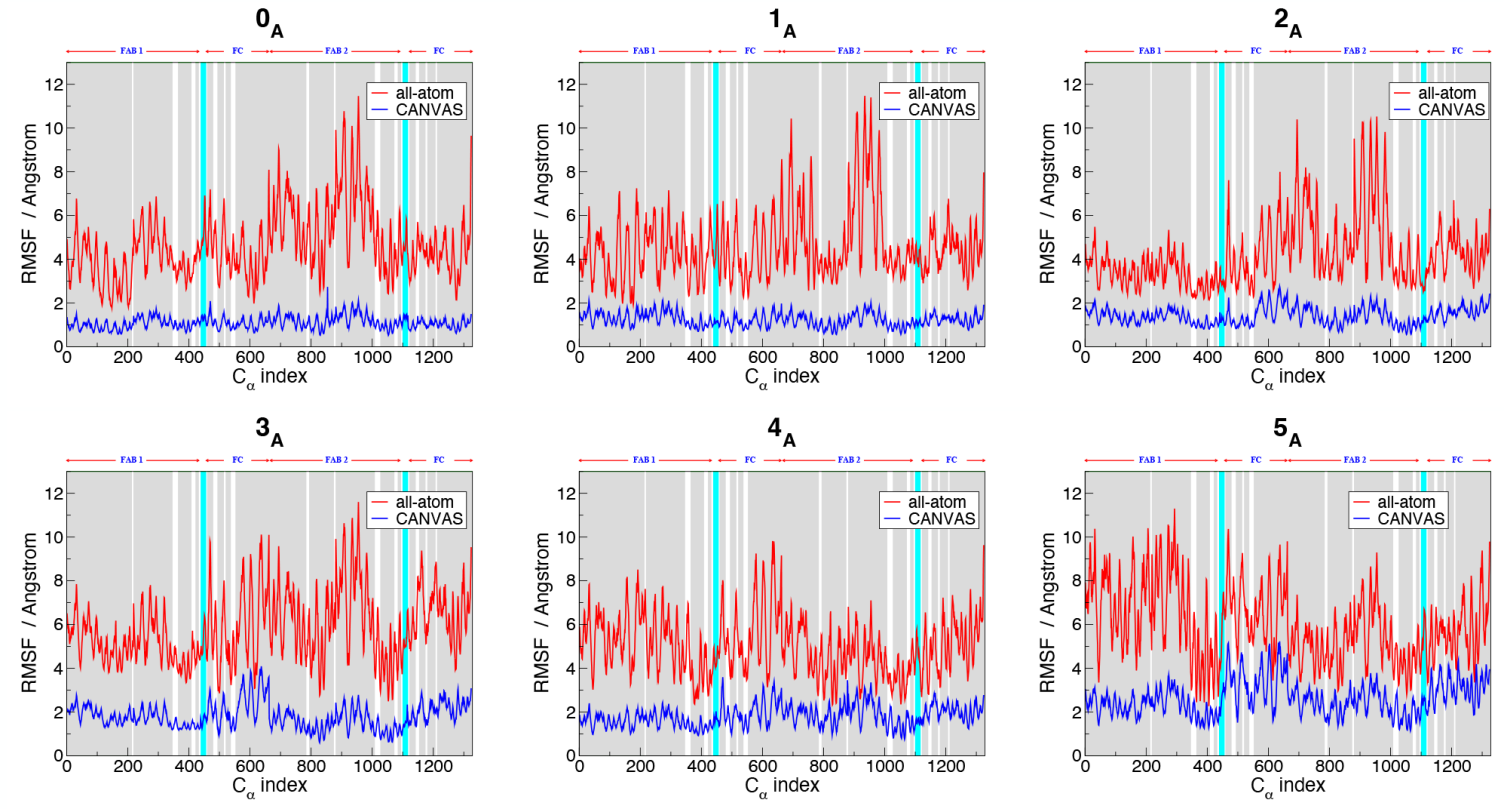
RMSF computed on C_α_ atoms of each apo form of pembrolizumab, for all-atom (red lines) and CANVAS simulations (blue lines). The cyan slabs correspond to the hinge region described atomistically in the CANVAS model, whereas the gray and white regions correspond to the parts of system modeled as CG and MG, respectively. The *x*-axis corresponds to the C_α_ indices.

The average residue fluctuations were evaluated by computing the RMSF of each C_α_ in the all-atom part and each CA bead (CA_mg_, CA_cg_) in the MG and CG ones (whose position is the same of the corresponding C_α_ atoms in the all-atom representation). The analysis of the RMSF plots (Figure 9) shows that, for each cluster, the fluctuations follow the same trend for both all-atom and CANVAS simulations; nonetheless, the RMSD and RMSF values of pembrolizumab in the CANVAS case appear rather low when compared to those of the ADK simulations, where the multiresolution model quantitatively reproduces the atomistic fluctuations. This may be ascribed to the interconnections between distant antibody regions, which take place through an extended network of interdomain correlations within and around the hinge.^60^ Indeed, we expect that the differences between atomistic and multiscale fluctuations are due to the particularly small high-resolution region chosen for the pembrolizumab with respect to ADK; in the latter case, ∼62% of the residues is described atomistically, whereas in the former case only ∼3% of the residues is at high resolution. To test this hypothesis, we performed an additional 50 ns long CANVAS simulation of pembrolizumab with a larger size of the atomistic region; here, the number of atomistic residues is about 16% of the total. The resulting RMSF (Figure S9) shows that including in the high-resolution region also those Fab and Fc residues that are in contact with the hinge region leads indeed to a better agreement between the all-atom and CANVAS simulations, with respect to the case where the hinge region alone is treated atomistically. In this regard, we stress that the choice of the optimal level and distribution of coarsening to be employed in the construction of a multiple-resolution protein model is a complex and difficult task *per se*;^9,50^ nonetheless, CANVAS would represent a powerful instrument to investigate precisely this aspect, in that it allows a simple parametrization of the model and the subsequent study of the optimal resolution modulation required to correctly and quantitatively reproduce specific system features.

Residue fluctuations are further investigated by computing the linear correlation between the RMSF of C_α_ atoms of fully atomistic simulation and the CANVAS one for each case. The latter is given by the calculation of the Pearson coefficient^79^ ρ, as reported in the scatter plots of Figure S10. All clusters show satisfactory results, with good RMSF correlations (ρ ∼ 0.7); moreover, an excellent correlation is found in cluster 0_A_ (ρ ∼ 0.87). To gain additional information about the latter result, we also calculated the *cross* Pearson coefficient ρ_*XY*_ between states and models as summarized in Table 3. *X* and *Y* take values in [0, 5], corresponding to the various clusters, whereas each variable is associated with the all-atom (*X*) and CANVAS (*Y*) model. For instance, the value of ρ_25_ corresponds to the Pearson correlation coefficient between the RMSF of C_α_ atoms for the 2_A_ cluster at fully atomistic resolution versus the RMSF of the 5_A_ state simulated with CANVAS. Diagonal elements ρ_*XX*_ measure the correlation between C_α_ atoms of a fully atomistic simulation and corresponding CANVAS one for the same cluster. Such values, already displayed in the scatter plot of Figure S5, are highlighted in bold in the table. One can notice that the higher the cluster index, the lower the value of the Pearson correlation coefficient (ρ_00_ = 0.87, ρ_55_ = 0.68). Because the clusters are ranked by increasing radius of gyration (or equivalently decreasing compactness), the reason for this trend can be ascribed to the fact that the CANVAS model of a more open structure has more freedom to explore conformations further and further away from the reference.

**Table 3:**
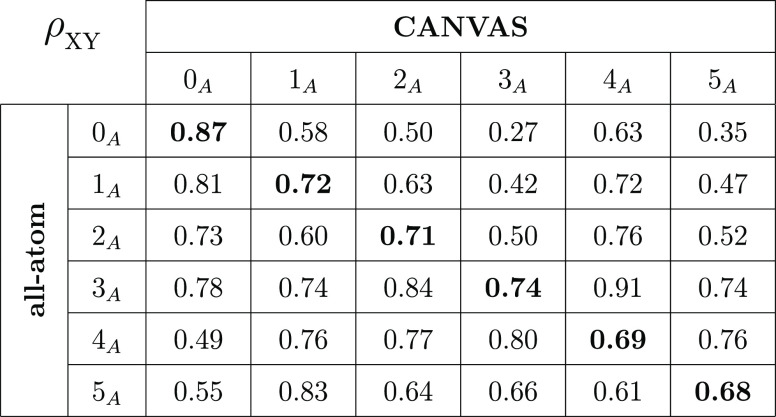
*Cross* Pearson Coefficients ρ_*XY*_ between States and Models^a^

a *X* and *Y* refer to the all-atom and CANVAS model, respectively; both indices correspond to the conformation from which the simulations start (0_A_, 1_A_, 2_A_, 3_A_, 4_A_, 5_A_). On the diagonal, the higher the index *XX*, the less compact the antibody conformation and the lower the value of ρ_*XX*_.

Furthermore, Table 3 shows that the Pearson coefficient is not systematically higher when comparing simulations starting in the same conformational basin. This is not a fully unexpected result; indeed, CANVAS simulations were started from the given initial conformations that in this case are also representative of specific groups of structures sampled in an all-atom MD trajectory, but this gives no guarantee that the whole run will explore the same cluster. This is true in general, even in the case of a fully atomistic model: a new all-atom simulation starting from a representative frame of one conformational cluster might, because of its stochastic nature, diffuse toward another cluster and hence show a fluctuation pattern closer to what is observed in a different set of frames. In the case under examination, additionally, the CANVAS model consists of a distinct structural representation and interaction force field with respect to the all-atom reference; hence, even if the simulation starts from a representative frame of the all-atom cluster, this frame will not be an equilibrium, representative configuration of the conformational space that would be sampled by the CANVAS model. What we observe in our analysis is that, in spite of this *caveat*, the CANVAS simulations show a remarkable structural overlap between the conformations sampled starting from a given frame and the all-atom cluster they represent, as can be seen from the CANVAS simulation trajectories provided as Supporting Information; as for the pattern of fluctuations, the strong intracluster consistency is paired by a non-negligible, and sometimes higher, correlation with different reference clusters, whose appearance is thus not unexpected nor surprising. Hence, although further work is certainly needed to perfect the agreement between the all-atom model and its multiple-resolution counterpart, the strong structural consistency and the highly correlated RMSF patterns of CANVAS run against their corresponding references support the idea that the model can already capture rather fine details of the molecule’s dynamics.

Further analyses were performed to differentiate the dynamics of all-atom and CANVAS simulations for different clusters. Specifically, we have examined the fluctuation correlations distinguishing residues by their level of resolution (AT, MG, CG) and the domain they belong to (FAB1, FAB2, FC). This analysis highlights other salient properties of the fluctuations of the antibody: (1) *Scatter plot with points colored based on resolution* (AT, MG, CG) in Figure S11. The all-atom part is very small (∼3%); hence, the corresponding value of the Pearson coefficient is not indicative. Conversely, the medium- and coarse-grained parts make up for most of the antibody (∼97%); hence, the value of ρ_MG_ and ρ_CG_ is closer to the one of the full system (dash black line). (2) *Scatter plot with points colored based on the domain partition* (FAB1, FAB2, FC) in Figure S12. Each domain produces a linear pattern in the plot, and the values of the corresponding Pearson coefficients is close to unity. It is worth noticing that, in some of the clusters, the RMSF of the two Fab domains indicates differences in flexibility between the all-atom and the CANVAS models. Specifically, although the overall correlation degree is rather high, the slope of this correlation is different between the two domains. A close inspection reveals indeed that the two heavy chains present a different arrangement of the hinge and the CH_2_ domain, as already noted elsewhere,^54,60^ thus returning a model whose Fab domains have different interactions and, therefore, different flexibilities.

These analyses provide an additional confirmation that the RMSF correlation between all-atom simulation and the CANVAS one is rather high, although more sophisticated and less straightforward than expected; this, in hindsight, is a reasonable behavior for a system whose structural and dynamical modules are represented, modeled, and simulated with distinct levels of resolution.

The conformational dynamics of the system was further inspected by computing the RMSIP between the essential subspaces given by the first *n* normal modes of the covariance from the atomistic and CANVAS simulations, with *n* ranging from 1 to the first 10 modes. A value of 0 indicates that the two mode subspaces are orthogonal, whereas 1 indicates that they are identical.^71^Figure S13 shows that less than 5 modes are enough to attain a very good overlap (RMSIP > 0.8) for all clusters.

We compared the similarity of the structures sampled in the atomistic and CANVAS simulations through the calculation of the radius of gyration (Figure S14). The values present small deviations, with the largest discrepancy of 1.3 Å observed in cluster 3_A_; however, in all cases, the radius of gyration from the all-atom simulations is slightly larger than that from the multiscale case, arguably because the steric effects of the side chains cannot be perfectly matched in the very coarse representation employed here, where only the C_α_ or backbone atoms are retained for more than 97% of the residues.

**Figure 10:**
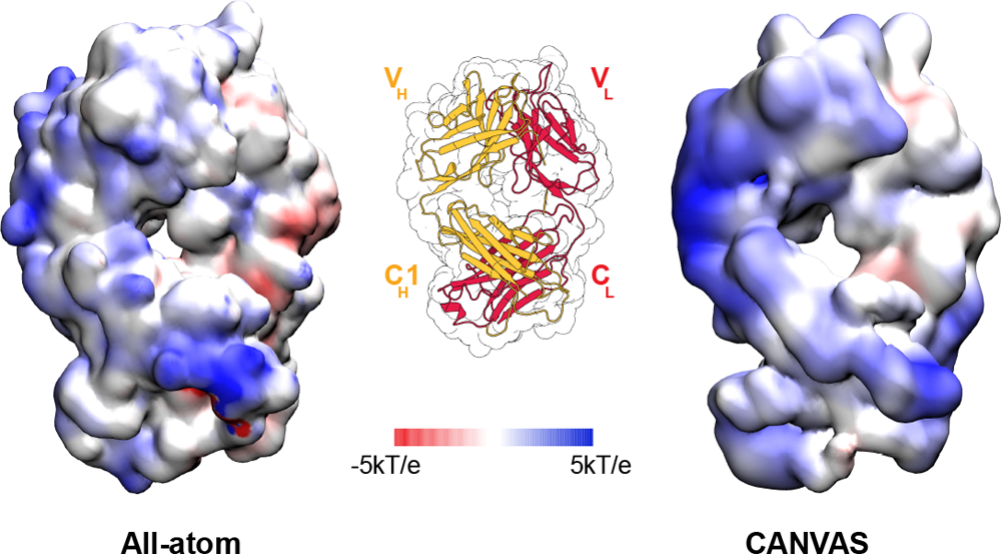
Electrostatic potential calculated with the APBS for the all-atom and CANVAS representations of pembrolizumab Fab1, mapped on the protein surface.

As previously done for ADK, the electrostatic potential of the Fab1 domain at MG/CG resolution has been computed for the antibody Fab, on the basis of the partial charges assigned to each bead in the CANVAS model (Figure 10). The comparison between the all-atom and low-resolution case shows a good similarity.

**Figure 11:**
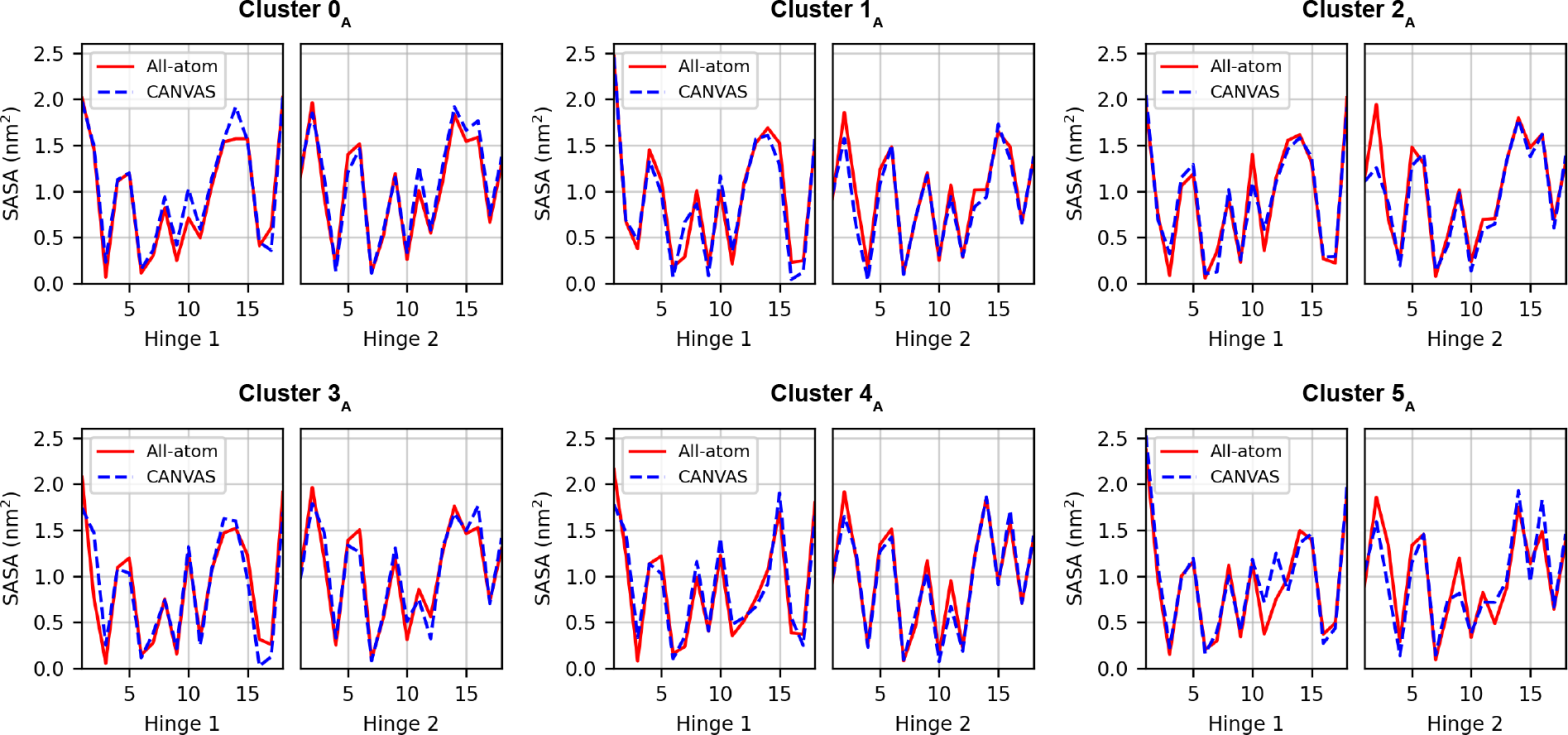
Per residue values of SASA, computed for the atomistic region of the antibody in each conformational basin. Hinge 1 belongs to chain B, whereas hinge 2 belongs to chain D.

The average SASA was computed along the trajectory for each residue of the atomistic region, namely, the two hinge segments (Figure 11). The comparison between the SASA values computed from the all-atom and multiscale simulations, performed for each conformational cluster, shows a very good agreement. The CANVAS model proves able to accurately reproduce the solvent exposure of the atomistic residues in relation to the conformational properties of the fully atomistic system.

## Conclusions

In this work we introduced the CANVAS model for the multiscale molecular dynamics simulation of proteins. The model couples different levels of detail within the same protein representation, ranging from a fully atomistic description to a coarse one, for example, one bead per amino acid (as in the case studies discussed here) or even lower levels of resolution. CANVAS allows a smooth transition between these resolutions by including regions at intermediate levels of detail. Importantly, the nonbonded components of the interaction potential are taken into account at all resolution levels by assigning to each CG bead the average properties—including charge, size, and dispersion energy—of the atoms that are mapped onto it. This property enables, in principle, the application of CANVAS for the simulation of large, multimeric protein complexes, where also the CG resolution can be used to model realistic molecular interfaces. This application will be explored in future works.

Here, we have tested the CANVAS model on two systems of very different size and conformational dynamics, namely, the enzyme adenylate kinase and the therapeutic antibody pembrolizumab. To validate the model, we performed a comparison among the properties extracted from the fully atomistic and multiscale simulations, in terms of residue fluctuations, large-scale dynamics, solvent exposure, and electrostatic properties; in all cases, the CANVAS model results are in good agreement with the all-atom reference.

The variable-resolution modeling approach presented here achieves two key goals: first, it demonstrates that a sensible modulation of the resolution can be employed to construct models of large molecules whose behavior is the same of, or quantitatively consistent with, that of a reference all-atom model of the system; second, it enables the rapid, practical construction of tailored low-resolution models of such molecules with minimal information and no reference simulations. The possible applications of these models cover a broad spectrum; we here stress those that appear most promising to us, namely, the exploration of the conformational space of molecules whose structure is known with low resolution only, or the characterization of the structure–dynamics–function relation by means of the systematic modulation of the resolution throughout the structure. An additional future application is the efficient calculation of binding free energies, employing an atomistic accuracy only in the active and/or allosteric sites. The relevance of taking into account distant protein domains within the simulation setup has been proven in various cases, as for example specifically observed in the case of antigen–antibody binding affinities;^60,80^ the possibility of keeping a simplified description of the vast majority of the molecule thus represents an advantage with respect to the alternative approach of simulating only the protein domain involved in the binding. In addition, we can expect that the impact on entropy due to the reduction of the number of degrees of freedom is similar, and therefore does not affect the result, if the aim is to compute relative binding free energy among a set of similar ligands, where the mapping of the protein is kept the same. All the above-mentioned applications, which involve the usage of the CANVAS model in combination with efficient simulation methods for the solvent (e.g., multiple-time-step,^81,82^ implicit solvent,^67,68^ or adaptive resolution simulation methods^32,33,69^) are currently under development and pave the way to a novel approach to computer-aided molecular biochemistry.

## Data Availability

The CANVAS software is available for download at https://github.com/potestiolab/canvas, including the manual and tutorials. The raw data produced and analyzed in this work are freely available on the Zenodo repository 10.5281/zenodo.7225086.

## Supporting Information Available

Derivation of the elastic constants *k*_nb_; RMSD and RMSF of ADK using GROMACS/LAMMPS and Charmm/Amber force fields; RMSF from atomistic simulations of ADK using reaction field or PME; distance between C_α_ atoms of residues A55 and V169 in the all-atom simulation of ADK; alignment of atomistic and CANVAS representative structures, in open and closed conformations; RMSF of ADK from atomistic and CANVAS simulations, in open and closed conformations; representative structures of pembrolizumab antibody; RMSD from pembrolizumab simulations; RMSF from pembolizumab simulations with different sizes of the atomistic region; scatter plots of RMSF for each pembrolizumab cluster, and calculation of Pearson coefficient; scatter plots colored on the basis of the resolution and of the structural domain; RMSIP between the essential subspaces computed from the atomistic and CANVAS simulations; average radius of gyration for each conformational cluster of the antibody (PDF)
