# Supplementary material for: Fast, Accurate, and System-Specific Variable-Resolution Modeling of Proteins
Source: J Chem Inf Model. 2023 Feb 3;63(4):1260–75. doi: 10.1021/acs.jcim.2c01311 (PMC9976289; doi:10.1021/acs.jcim.2c01311)
Supplement: Supplementary file 1 — ci2c01311_si_001.pdf [file ci2c01311_si_001.pdf]

Supporting Information for: Fast, Accurate, and System-Specific Variable-Resolution Modelling of Proteins

Raffaele Fiorentini[1,2], Thomas Tarenzi[1,2], Raffaello Potestio[1,2,*]

[1] *Department of Physics, University of Trento, via Sommarive 14, I-38123 Trento, Italy*

[2] *INFN-TIFPA, Trento Institute for Fundamental Physics and Applications, via Sommarive 14, I-38123 Trento, Italy*

* Email address: raffaello.potestio@unitn.it

S1 Derivation of the elastic constants k_{nb}

As discussed in the main text, in the CANVAS model two non-consecutive C_α beads—be they part of "medium" or "coarse grained" residues as prescribed by the CANVAS scheme—are connected *via* a harmonic potential of stiffness k_{nb} if their distance in the reference (native) conformation of the system lies within a cutoff of 1.4 nm. Importantly, the magnitude of k_{nb} decreases as the distance d between the pair of C_α's increases; in this section, we will describe all the technical details involved in the parameterisation of $k_{nb}(d)$.

Firstly, we extracted from the all-atom MD simulations of pembrolizumab [1] a subset of the probability distributions $P_{ij}(r_{ij})$ of the scalar distance $r_{ij} = |\mathbf{r}_i - \mathbf{r}_j|$ between two non-consecutive α-carbon atoms $C_{\alpha,i}$ and $C_{\alpha,j}$, where $i, j = 1, ..., N_R$ run over the N_R total residues in the system. This analysis was conducted separately for each chain composing the macromolecule, and further restricting the set of possible $C_{\alpha,i}$—$C_{\alpha,j}$ pairs to all those whose separation along the sequence satisfies $1 < j - i < 16$—thus neglecting consecutive residues. Starting from the set of $P_{ij}(r_{ij})$, we then relied on the direct Boltzmann inversion method [2] to determine an effective, harmonic pair potential $V_{ij}(r) = \frac{1}{2}k_{ij}(r_{ij} - r^0_{ij})^2$ acting among each pair of C_α sites. The associated harmonic constants k_{ij}—actually, the combination βk_{ij}, with $\beta = 1/k_B T$—and equilibrium distances r^0_{ij} where obtained by separately fitting each $P_{ij}(r_{ij})/r^2_{ij}$ to a Gaussian distribution.

Distal residues along the sequence are not necessarily distant in the three dimensional conformation of the system. As such, the resulting ensemble of harmonic constants βk_{ij} was then binned according to the spatial distance d between the associated α-carbon atoms in the reference (native) conformation of the antibody. The distance-dependent spring constant $\beta k_{nb}(d_n)$ at distance d_n was then determined as the average

of all the βk_{ij} contained in bin $[d_n, d_n + \Delta]$, with $\Delta = 0.1$ nm and on an overall interval $[d_{min}, d_{max}] = [0.4, 4.5]$ nm.

The results obtained for $\beta k_{nb}(d)$ are presented in Figure S1. We note that $\beta k_{nb}(d)$ displays a steep decrease for short separations, gradually moving towards a plateau for increasing distance between the two C_α beads. Importantly, although we determined $\beta k_{nb}(d)$ up to $d_{max} = 4.5$ nm, we again stress that in the CANVAS model two C_α beads are connected by a weak spring only if their distance in the native conformation is smaller than 1.4 nm. Finally, to avoid, in the force field parameters of CANVAS, the presence of abrupt changes in the weak spring constant k_{nb} for slight variations of the distance, the data for $\beta k_{nb}(d)$ were fit to a power law $f(d) = \gamma + \delta d^\alpha$, finding $\gamma = 81.22$ nm^{-2}, $\delta = 1322.06$ nm^{-2}, and $\alpha = -1.26$. Results for the fit are included in Figure S1, and display that the fitting function captures the overall behavior of the raw $\beta k_{nb}(d)$ data points within the region relevant for CANVAS, that is, for $d \in [0.4, 1.4]$ nm.

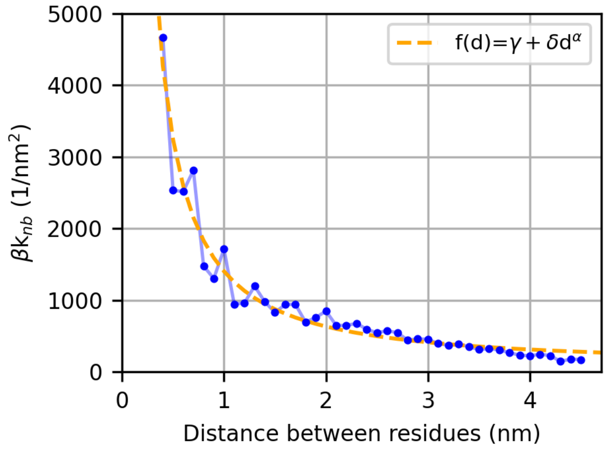

Figure S1: Dependence of the harmonic constants with respect to the spatial distance between C_α atoms. Data are fitted with the orange dashed curve, as described in the text.

S2 Supplementary figures

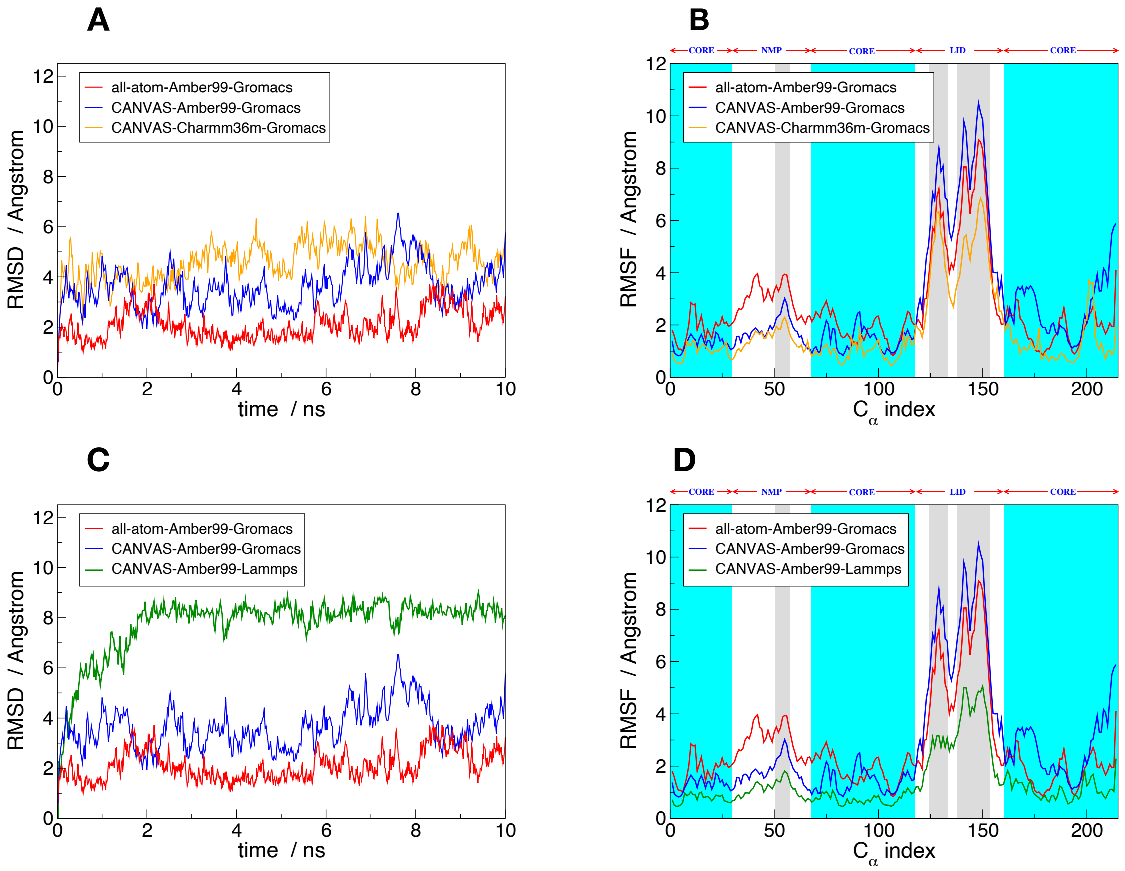

Figure S2: RMSD and RMSF from the all-atom and CANVAS simulations of ADK performed with GROMACS software and the Amber99sb-ildn force field, compared with results from the CANVAS simulations performed with LAMMPS using the same force field, and with GROMACS using CHARMM36m force field. The latter simulations have been acquired for 10 ns. The cyan areas in plots B and D correspond to the CORE domain, which is described atomistically. The high RMSD values of the CANVAS Amber-LAMMPS simulation (plot C) are compatible with the compact ADK conformation, which is reached after a few nanoseconds of simulation.

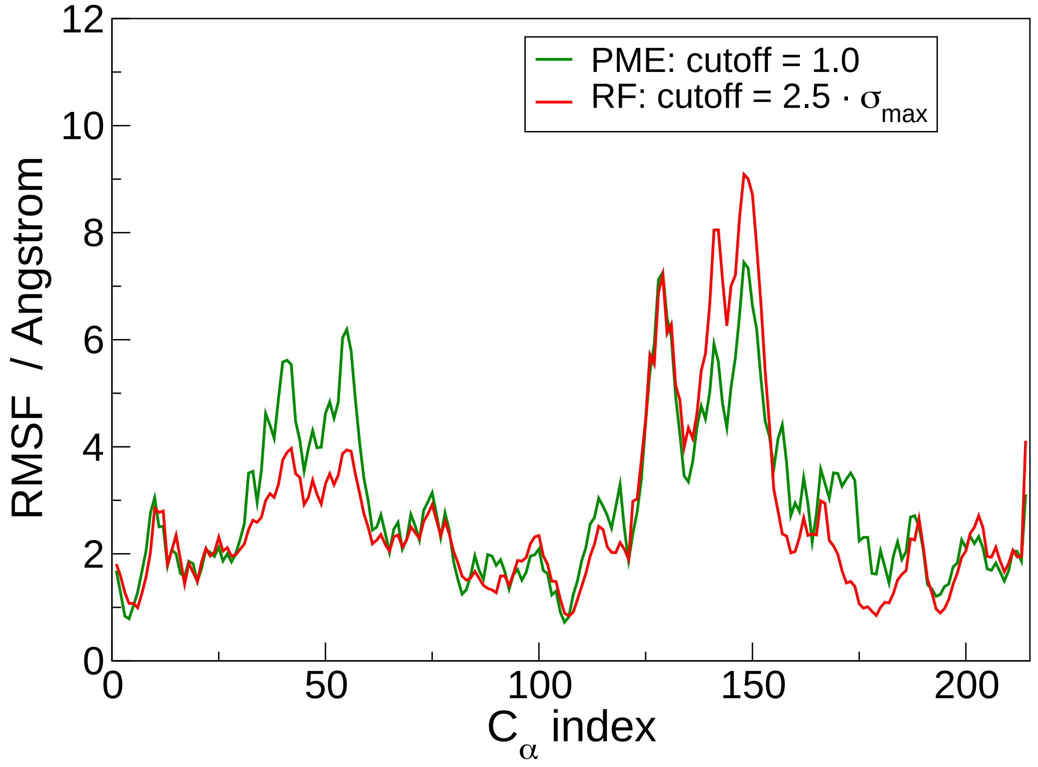

Figure S3: RMSF for each C_α of ADK from the all-atom simulation using the reaction-field method with a cutoff of 2.5 σ_{max} (red line) and PME method with a cutoff of 1.0 nm (green line). We can observe that the trends of C_α fluctuations are consistent with each other, providing evidence that AMBER can be safely employed with the reaction-field electrostatic method.

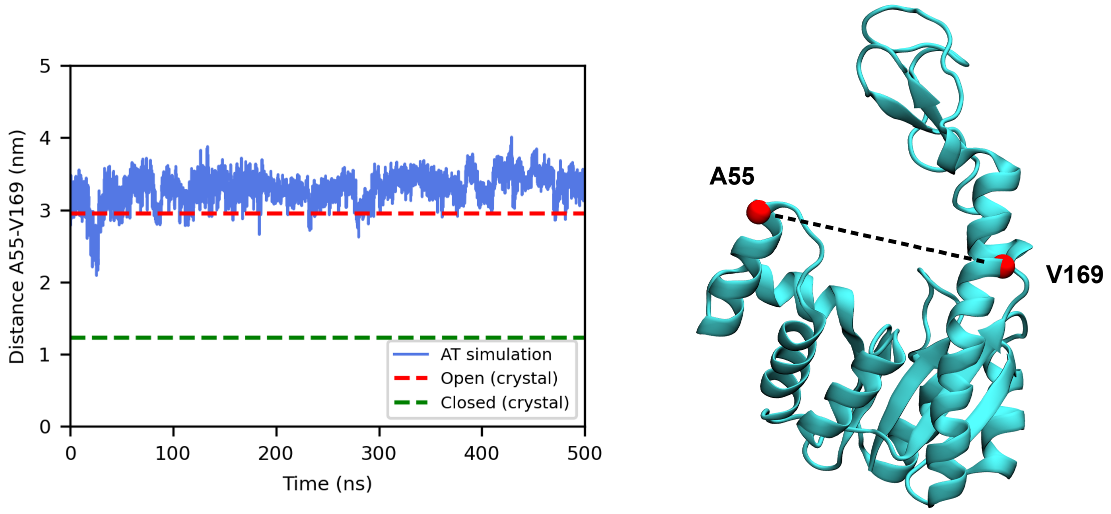

Figure S4: Distance between C_α atoms of residues A55 and V169 in the all-atom simulation of ADK. Values found in the crystal structure of the open and closed conformations (PDB IDs 4AKE and 1AKE, respectively) are also reported.

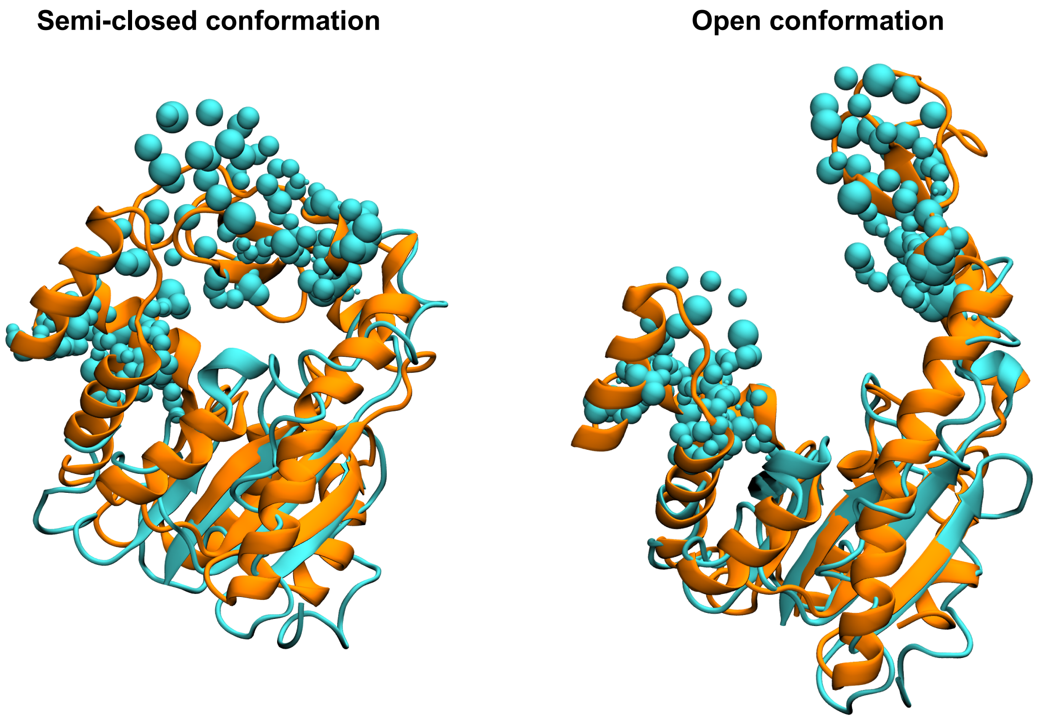

Figure S5: Alignment of the representative compact and open conformations of ADK, from the atomistic (orange) and CANVAS (cyan) simulations. The two sampled conformational states appear very similar; differences are observed mainly in the local arrangement of loops and flexible regions.

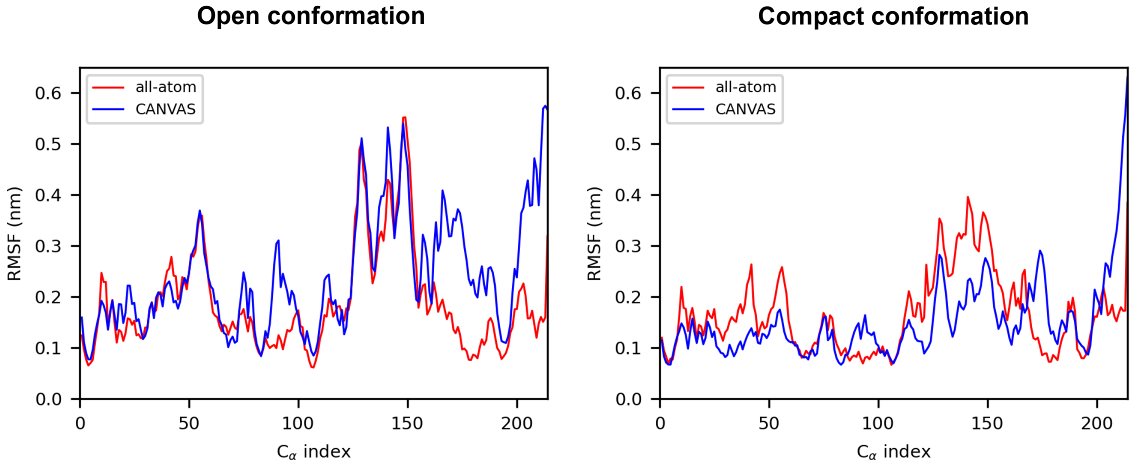

Figure S6: Root-mean-square fluctuations (RMSF) of C_α atoms from all-atom and CANVAS simulations of ADK, after clustering of the simulation frames on the basis of the RMSD and identification of two main conformational states (open and compact).

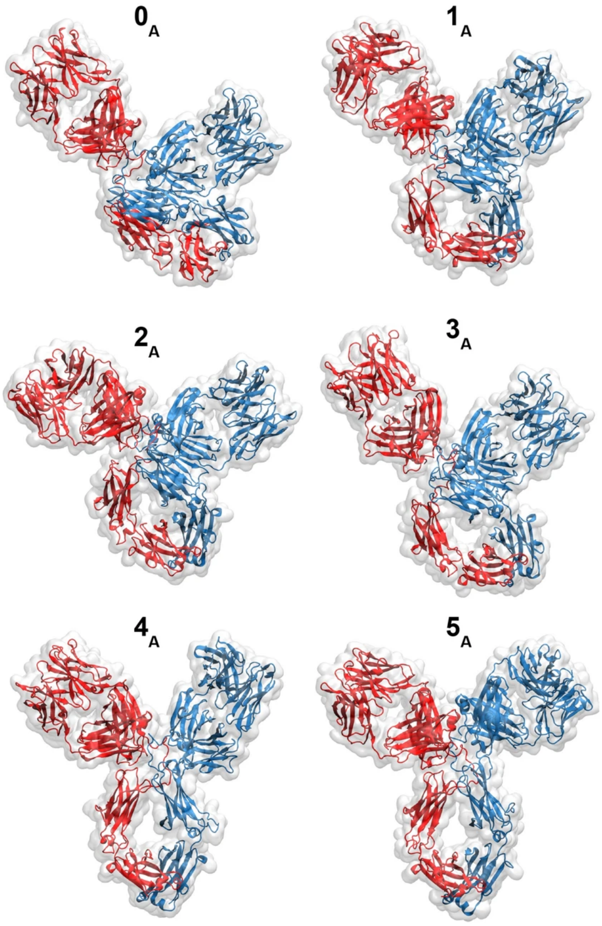

Figure S7: Representative structures of deglycosilated pembrolizumab in the apo form, for each conformational cluster. Chains A and B are in blue, and chains C and D in red. Adapted with permission from ref. [1]. Copyright 2021, The Authors.

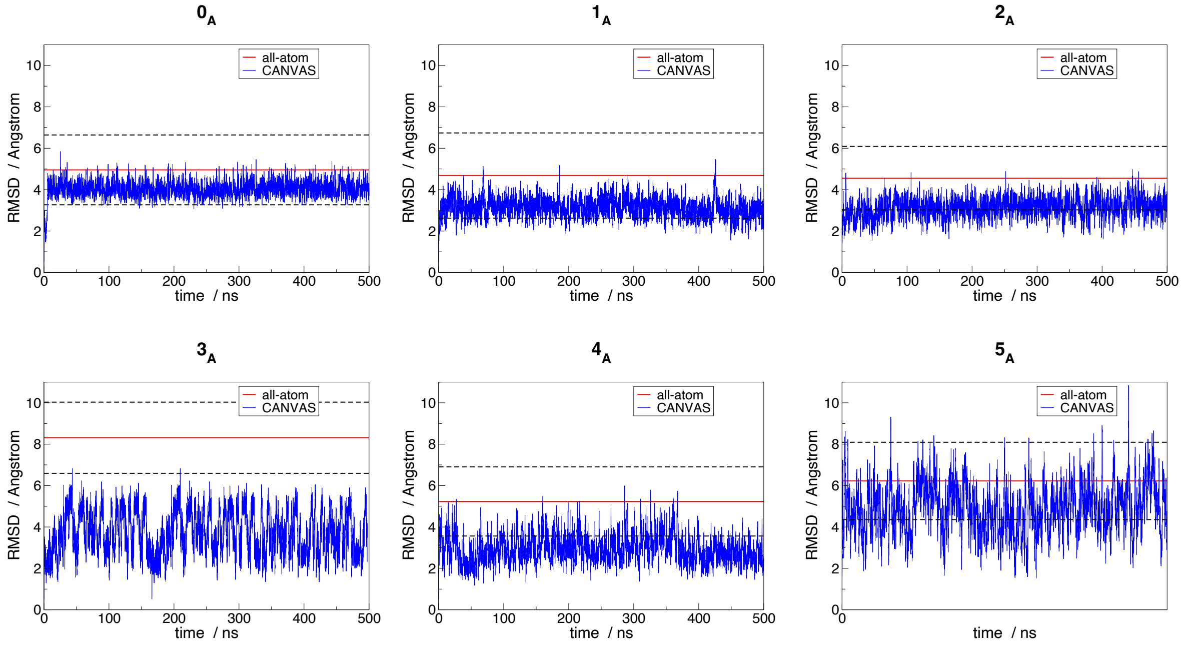

Figure S8: RMSD of C_α atoms comparing the all-atom simulations (red line) and the CANVAS ones (blue line). In all cases, the RMSD is computed with respect to the representative conformation of the cluster. The atomistic RMSD is represented as the average value (together with the corresponding standard deviation, dashed lines), since it is computed from atomistic frames that have been clustered on the basis of the protein conformation, and are therefore not temporally consecutive. The average CANVAS RMSD is generally slightly lower than the all-atom one; however, the former agrees with the atomistic reference within the error bar in all cases, except for the cluster 3_A. It is worth noting that the various clusters feature very close values of average RMSD both within the CANVAS model and the all-atom simulations; cluster 3_A represents an exception in the latter case, being appreciably higher than the others.

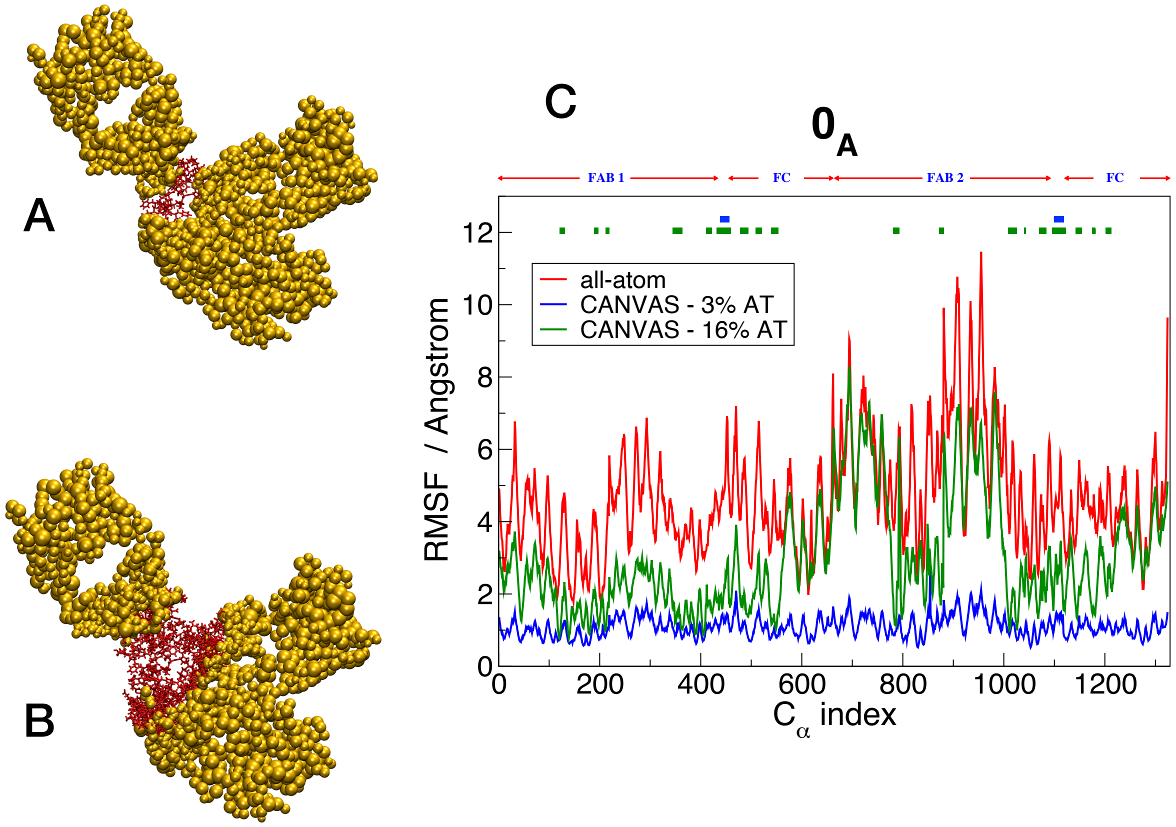

Figure S9: (A) and (B): Representative conformation of pembrolizumab from the 0_A cluster, where the atomistic residues (in red) constitute the 3% and 16% of the total, respectively (the medium- and coarse-grained regions are represented with yellow ocher beads). (C) Root mean square fluctuation (RMSF) computed on C_α atoms of structures obtained from the 0_A cluster (in the all-atom case, red line) or from simulations starting from this cluster's reference structure (in the CANVAS case). CANVAS simulations employ a number of atomistic residues equal to 3% and 16% of the total (blue and green lines, respectively); the duration of these simulations is 200 ns and 50 ns, respectively. The regions described atomistically in the CANVAS simulations are indicated with blue and green rectangles on the top of the plot. It is possible to notice that, by increasing the atomistic region, the trend of fluctuations gets closer to the atomistic reference.

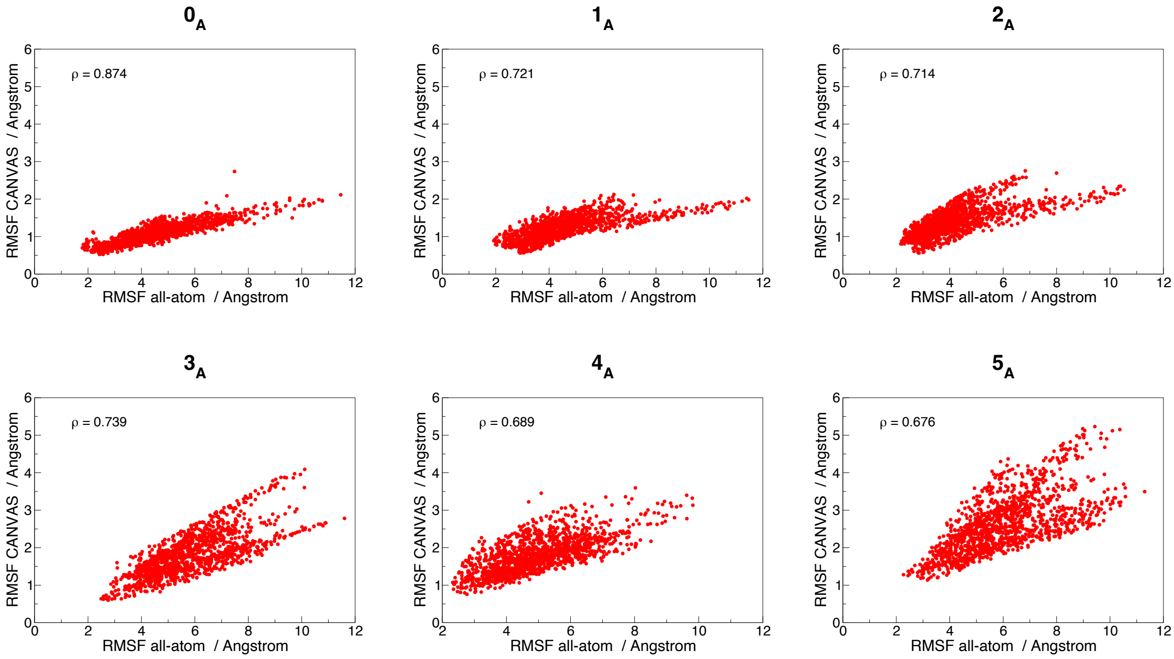

Figure S10: Scatter plots of RMSF for each apo form of pembrolizumab, comparing the results of all-atom simulation and CANVAS one. ρ indicates the value of Pearson Coefficient computed between the two sets of fluctuations. All clusters show satisfactory results: an excellent correlation is found in 0_A cluster ($\rho \sim 0.87$); the other clusters present good RMSF correlations ($\rho \sim 0.7$).

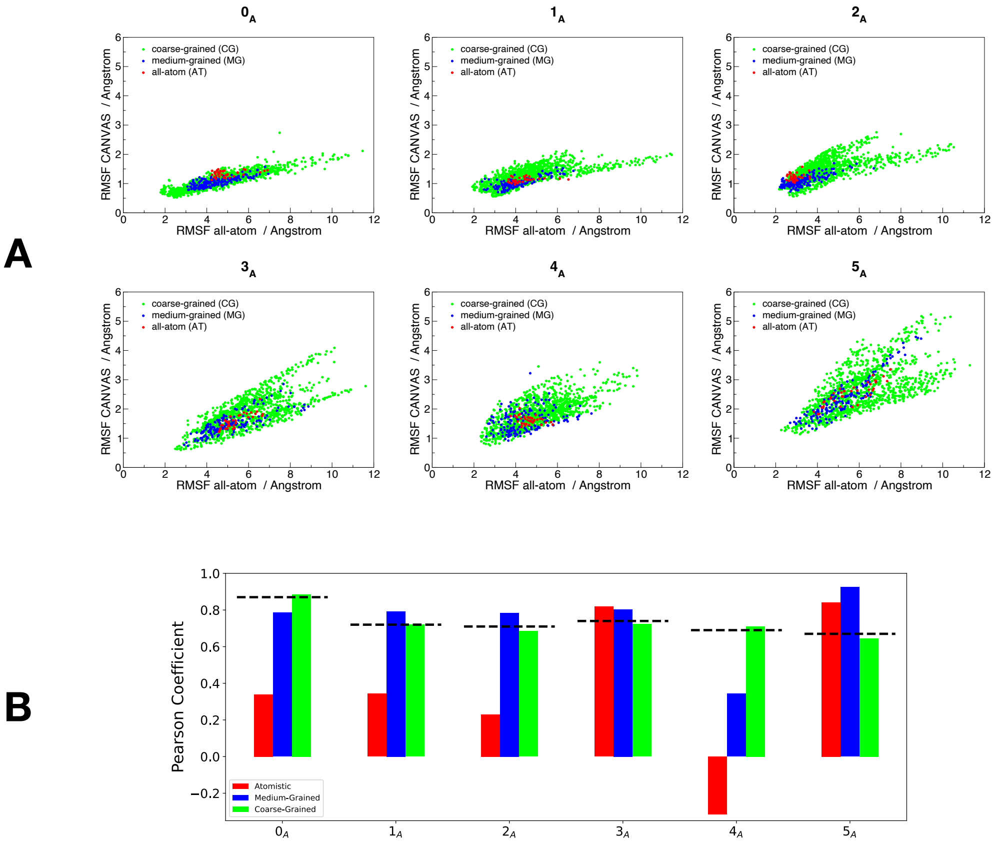

Figure S11: (A) Scatter plots of RMSF for each apo form of pembrolizumab, with points colored based on resolution (AT, MG, CG), comparing the results of all-atom simulation and CANVAS one. In particular, green, blue and red points correspond to coarse-grained, medium-grained and all-atom resolutions of CANVAS model, respectively. We notice that the all-atom part comprises a small region of the system ($\sim 3\%$ of the number of residues). (B) histogram of pearson coefficients, for each apo form, split according to the resolution ($\rho_{\rm AT}$, $\rho_{\rm MG}$, $\rho_{\rm CG}$). The dash black line corresponds at the value of ρ when considering the system in its entirety. We can notice that, except for clusters 3_A and 5_A, the pearson coefficient related to the atomistic part ($\rho_{\rm AT}$) is generally lower than the reference one; moreover, for 4_A cluster, $\rho_{\rm AT}$ is a negative number, meaning that atomistic and CANVAS C_α atoms are slightly *anti-correlate*. This behavior can be explained by the fact that the atomistic region is disordered and, as such, its fluctuations are *per se* less reproducible than those of the structured domains.

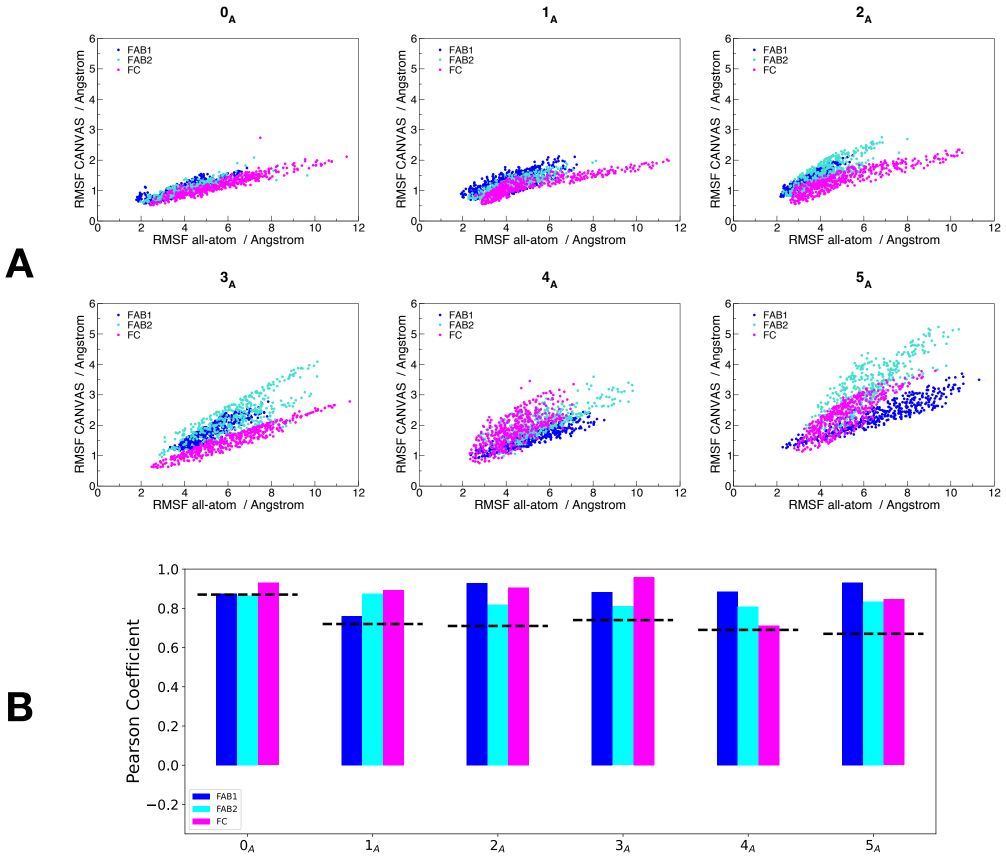

Figure S12: (A) Scatter plots of RMSF for each apo form of pembrolizumab, with points colored based on domain division (FAB 1, FAB 2, FC) comparing the results of all-atom simulation and CANVAS one. In particular, blue, cyan and magenta points correspond to FAB 1, FAB 2 and FC domains of system, respectively. From 0_A to 5_A the radius of gyration of reference structure increases, thus its compactness decreases accordingly and the CANVAS model is more likely to explore other conformations. Basically, it is evident that the scatter plot coloured according to the distinct structural domains in which the molecule can be decomposed shows clear linear patterns: each domain takes a linear region in the plot. (B) histogram of pearson coefficients, for each apo form, split according with the domain division (ρ_{FAB1}, ρ_{FAB2}, ρ_{FC}). The dash black line corresponds at the value of ρ when considering the system in its entirety. We can notice that, generally, the value of ρ is higher than the reference one for each domain, very close to 1. These results suggest that the overall RMSF scatter plot pattern can be better rationalised in terms of the fundamental dynamical substructures of the system, each of which shows its own correlation trend.

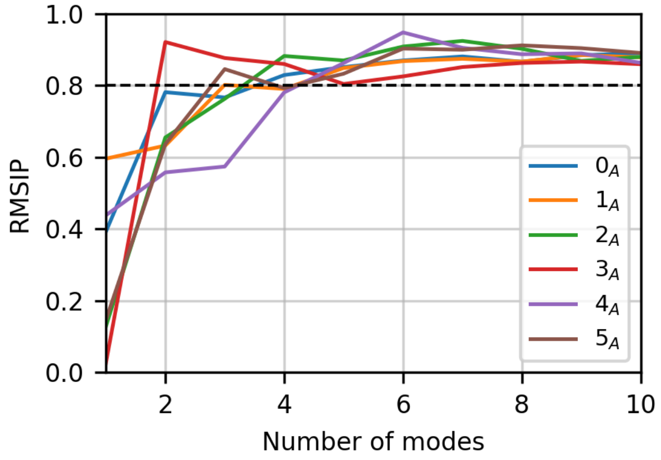

Figure S13: RMSIP between the essential subspaces computed from the atomistic and CANVAS simulations. In all clusters, 5 modes (or even less) are sufficient to reach an RMSIP larger than 0.8, indicating a very good overlap.

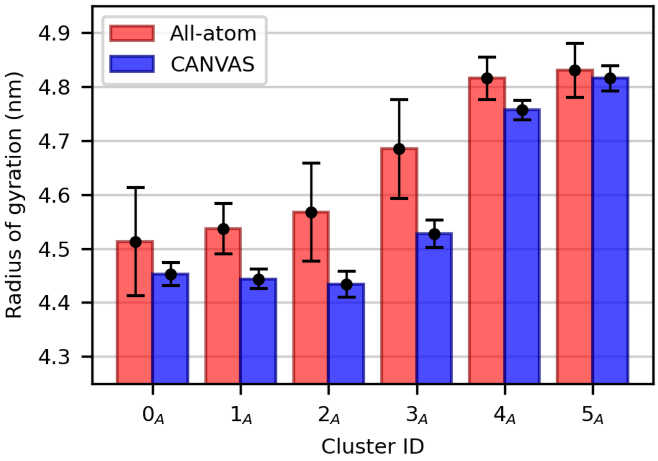

Figure S14: Average radius of gyration computed for each conformational cluster of the antibody, in the all-atom and multiscale representations. In both cases, only the positions of C_α atoms and CA beads were taken into account. Data for the all-atom case from ref. [1].

References

[1] T. Tarenzi, M. Rigoli, and R. Potestio, "Communication pathways bridge local and global conformations in an IgG4 antibody," *Scientific reports*, vol. 11, no. 1, pp. 1–12, 2021.

[2] D. Reith, M. Pütz, and F. Müller-Plathe, "Deriving effective mesoscale potentials from atomistic simulations," *Journal of computational chemistry*, vol. 24, no. 13, pp. 1624–1636, 2003.
